# Persistent radical anions in the series of *peri*-arylenes: broadband light absorption until far in the NIR and purely organic magnetism

**DOI:** 10.1007/s00706-019-02404-8

**Published:** 2019-05-02

**Authors:** Heinz Langhals, Ulrike Ritter-Faizade, Philipp Stadler, Marek Havlicek, Alexander Hofer, Niyazi Serdar Sariciftci

**Affiliations:** 1 Department of Chemistry, LMU University of Munich, Butenandtstr. 13, 81377 Munich, Germany; 2 Linz Institute of Organic Solar Cells (LIOS) and Institute for Physical Chemistry, Johannes Kepler University Linz, Altenbergerstrasse 69, 4040 Linz, Austria; 3 Department of Primary Nanometrology and Technical Length, Czech Metrology Institute, Okruzni 31, 638 0 Brno, Czech Republic

**Keywords:** Perylenes, Photochemistry, Radical anions, Fluorescence, Absorption, Electron spin resonance

## Abstract

**Abstract:**

Stable radicals in organic conjugated molecules are of great interest due to their magnetic signals and broad optical absorptions. In this paper, we report on naphthalene, benzoperylene, perylene, terrylene, and quaterrylene carboximides, reduced under controlled conditions, where stable metal-free solid salts of radical anions could be obtained forming darkly colored solutions with line-rich UV/Vis/NIR spectra and exhibiting special magnetic properties. The most bathochromic shift of the absorption maxima extend from 760 until 1700 nm. Persistent paramagnetic properties of the solids were observed and temperature-dependent susceptibilities are measured.

**Graphical abstract:**

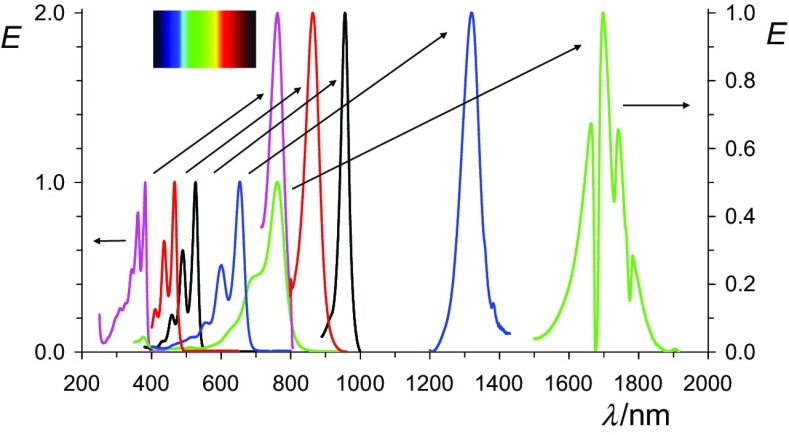

## Introduction

Absorption edge of organic compounds is in the ultraviolet (UV) because of large energetic gaps between the highest occupied molecular orbital (HOMO) and highest occupied molecular orbital (LUMO). Shifting light absorption edge into the visible requires molecular design and band gap engineering [1]. An extended π-system increases the absorption wavelength to obtain colored materials with lower energetic gaps [2–4]. On the other hand, smaller differences in energy levels are expected within the energetic band structure of the occupied and the unoccupied *π*-orbitals of complex aromatic and heteroaromatic systems. Thus, the removing or addition of one electron to an extended *π*-system would make these energetic sublevels of π-band and π*-band, respectively, accessible for electronic transitions to enable light absorption even until the near infrared (NIR) region. These may be extended far into the IR for sufficiently large electronic systems. However, there is an inherent tendency of such free radicals for dimerization recombination. This may be counteracted by the introduction of charge because of electrostatic repulsion between the molecules. We prefer radical anions avoiding the combination of the electrophilic radical cations and aromatics. As a consequence, suitable electron deficient structures are required for the uptake of electrons.

The *peri*-arylenebiscarboximides [5] **1** according to Scheme 1 are known for *n* = 1 absorbing in the UV [6, 7] to *n* = 4 and, finally, *n* = 6 [8] absorbing in the NIR and are attractive starting materials for the preparation of radical anions **2** because of their high stability, electron depletion by four carbonyl groups and broad region of tuning of light absorption.

**Figure:**
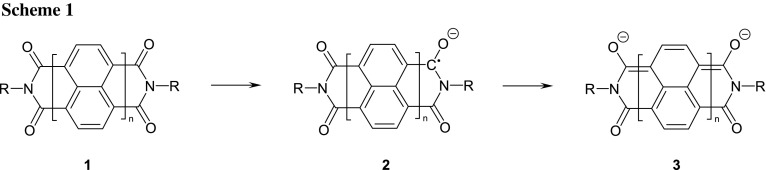

The radical anions **2** (*n* = 1) of the naphthalenebiscarboximides **1** (*n* = 1) were many times reported as intermediates in photochemical processes. The electrochemical reduction of derivatives of **1** (*n* = 1) to **2** (*n* = 1) was indicated by ESR signals [9]. The same method allowed the preparation of a dark crystalline electrically conducting, however, not further characterized material [10] attributed to **2**; this material seemed to be insoluble in polar aprotic solvents because of washing with acetonitrile was described. UV/Vis/NIR spectra of electrochemically prepared **2** were indicated [11]. Chemical reduction of **1** (*n* = 1) was described by means of strongly alkaline dithionite [12] solution without further characterization. UV/Vis spectra of radical anions **2** were obtained by flash photolysis and spectroscopically compared [13] with the products from reduction with dithionite and with cobaltocene [14]. A most bathochromic absorption at 777 nm in dichloromethane can be taken from the graph of the published spectra for the latter. Finally, cyclic triradical trianions were described [15–17].

## Results and discussion

Here, we studied the reduction of the naphthalenebiscarboximides **1** (*n* = 1) for the synthesis of the radical anions [18] **2** on a preparative scale and applied hydroxyacetone in alkaline media (NaOH) as an efficient, versatile reducing agent where there are well-defined products of the oxidation of the reductone. The preparation of the radical anions **2** (*n* = 1) was successful in various solvents for both for *R* = alkyl and *R* = aryl and was indicated by the bathochromic UV/Vis absorption with a maximum at 761 nm corresponding to the absorption of the electrochemically prepared radical anions.

**Figure:**
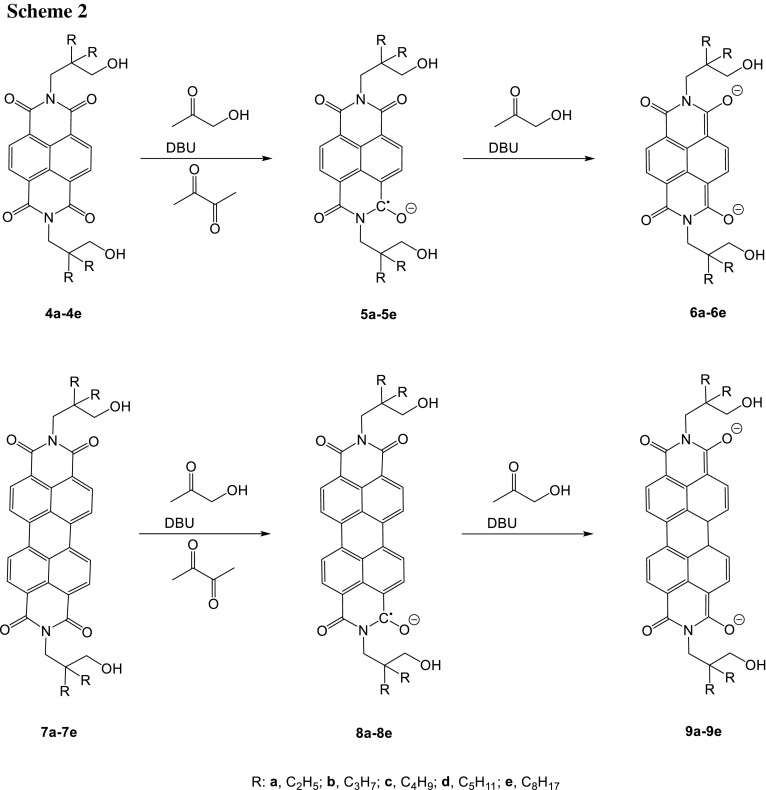

**Fig. 1:**
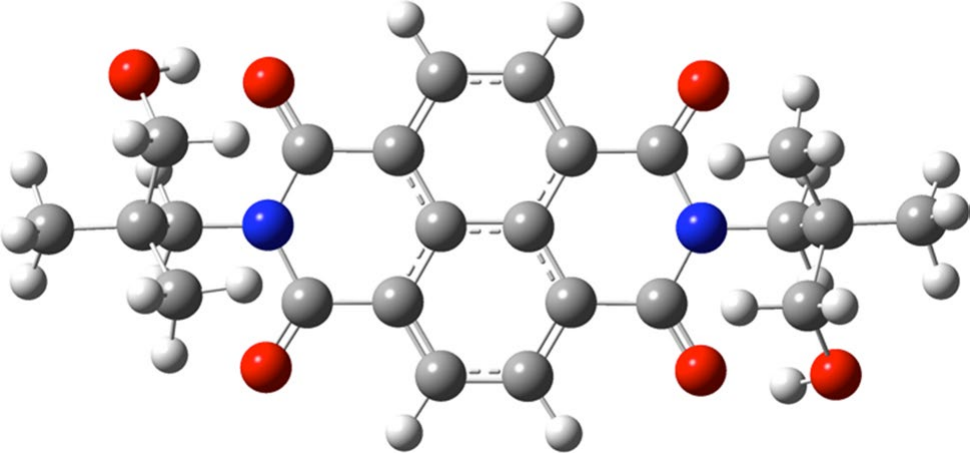
Quantum chemical (DFT B3LYP) calculated structure of **4** with R=CH_3_ (one rotamer is shown); the proximity of the OH and carbonyl groups can be clearly seen

However, three topics still remain for the improvement of the synthesis: (i) Over reduction in the redox equilibrium proceeds to the bis-anions **3** (*n* = 1) under these conditions and is indicated by an absorption at 612 nm (overlapping with the absorption of **2**). The ratio of **2** and **3** can be controlled by solvent effects where **3** dominates in ethanol, **2** exceeds in acetone, and about a 1:1 mixture of **2** and **3** is found in toluene. The equilibrium can be shifted to **2** by the addition of diacetyl as a weak oxidant corresponding to the oxidized hydroxyacetone. Thus, solutions can be obtained essentially containing **2**. (ii) The required alkaline medium causes slow hydrolysis of carboximides limiting the stability of solutions; hydrolysis is inhibited if R means a 3-hydroxypropyl group as was found in preceding work [19]; see Scheme 2. Thus, the series of compounds **4** proved to be very resistant concerning alkaline hydrolysis. For example, the refluxing of **4b** or **4e** with 23 equivalents of 85% KOH in *tert*-butylalcohol for 6 h was not successful for hydrolysis, but only caused slow ring contraction [20] where **4e** seems to react even more slowly. The formation of intramolecular hydrogen bonds with a more rigid structure may be, therefore, responsible and are indicated as well by the ^1^H NMR coupling of the hydroxy hydrogen atoms to form triplets excluding a fast intermolecular proton exchange as sharp bands for the OH groups between 3480 and 3550 cm^−1^. A slight shift of the carbonyl absorption of naphthalimides with aliphatic substituents at about 1705 and 1666 cm^−1^ by 5 cm^−1^ to lower wavenumbers may be a further indicator. Such intramolecular hydrogen bonds to the carbonyl groups can be verified by means of quantum chemical calculations as shown in Fig. 1.

The application of the non-ionic base DBU (1,8-diazabicyclo[5.4.0]undec-7-ene) [21–23] instead of NaOH is further favorable for a long-term stability the system. (iii) The solubility of **4** is limited constricting general applications and should be enhanced; as a consequence, we introduced geminal long-chain alkyl groups in **4a** to **4d** for increasing solubilization according to Ref. [24].

Considering topics (i)–(iii), we could prepare stable solutions of **5a**–**5e** in ethanol. We precipitated the radical anion by means of an isoionic addition in excess of a salt. Counterions of minor importance for solutions of **5**, however, fundamentally influence [25] the properties of solid materials. Best results for precipitation were obtained with tetrabutylammonium bromide where the addition of diacetyl proved to be not necessary because the lower solubility of salts of the radical anions **5** compared with the bis-anion **6** allows its selective precipitation along with a shift of the redox equilibria. The solid tetrabutylammonium salts of **5** are black solids stable at room temperature for years, if atmospheric oxygen is rigorously excluded; even a brief handling in air is possible because re-oxidation proceeds only slowly. In the IR spectrum, the carbonyl absorption of **5** between 1621 and 1632 cm^−1^ and for the second band between 1580 and 1587 cm^−1^ are appreciably shifted to lower wavenumbers compared with **4** and can be taken as an indicator for a weakening of the C=O– double bond by the delocalization of the unpaired electron in **5**. Even the C=C– vibration was shifted to lower wavenumbers and found at 1556 and 1520 cm^−1^. In the ^1^H NMR spectrum the signals of the tetrabutylammonium cation could be observed and signals from the periphery of the anion whereas the resonances of the aromatic protons could not be detected; this was attributed to appreciable line broadening and can be taken as a proof for the unpaired electron in **5**. A further proof was given by high-resolution anion mass spectroscopy. The solid radical salts can be re-dissolved to obtain reddish brown solutions containing exclusively the radical anion. Such solutions can be stored without decomposition for many months if atmospheric oxygen is rigorously excluded (handling becomes simplified by a stabilization with hydroxyacetone and DBU concerning traces of oxygen). Obviously, disproportionation is kinetically inhibited.

The reduction of perylenes **1** (*n* = 2) was studied as the next higher homologues of the *peri*-arylenes. Radical anions of perylenebiscarboximides **2** (*n* = 2) were firstly described [26] in 1978 and electrochemically generated [27–29] by the reduction of **1** (*n* = 2). A further electron depletion by means of trifluoromethyl groups allowed the isolation of salts of radical anions [30–34]. In this work, the generally pigment-like perylenecarboximides **1** (*n* = 2) were solubilized by means of long-chain *sec*-alkyl groups such as the 1-hexylheptyl group at their nitrogen atoms and reduced with hydroxyacetone in alkaline solution to form **2** (*n* = 2): Mostly over-reduction to the bis-anion **3** (*n* = 2) and slow hydrolysis of the carboximide groups proceeded. Here, we applied the same strategy for the preparation of the more stabilized **8** as was described for **5**. Thus, perylene tetracarboxydiimides **7** were protected against hydrolysis [19] with 3-hydroxyalkyl groups analogously to **4**, and reduced with hydroxyacetone and NaOH to form surprisingly stable products which are now the focus of the following discussion. Over reduction proceeded for **7a**–**7e** under these conditions to mainly form the bis-anions **9a**–**9e**, whereas only some radical anions **8a**–**8e** are found in the equilibrium; this may be a consequence of the electron-withdrawing effect of the hydroxy groups in the side chains of **7** stabilizing the higher charged bis-anion. The equilibrium can be shifted to the radical anions **8** by the addition of the weak oxidant diacetyl. The radical anions **8** can be precipitated from the reduced solution by the addition of tetra-*n*-butyl ammonium bromide. The preferred precipitation of the **8** compared with the bis-anions **9** may be a consequence of the more efficient lowering of the solubility by the isoionic addition of the ammonium salt for **8** than for the bis-anions (compare the reduction of **4**). The precipitated radical anions **8** form bluish dark solids and seemingly can be stored indefinitely under protective atmosphere such as argon. **8a** and **8b**, respectively, can be even briefly handled in air; however, slow oxidation proceeds to convert the substance back to the staring materials **7a** and **7b**, respectively. Essentially, solutions of the radical anions **8** are obtained on re-dissolution of the solids being comparably stable; disproportionation of **8** to **7** and **9** seems to be kinetically inhibited. The re-oxidation of the dissolved **8** with air to the starting material **7** proceeds much more quickly than for the solids. A trapping of the dissolved anions **8** by electrophiles such as protons, acetic anhydride or trimethylsilyl chloride was not successful because the starting materials **7** were spontaneously formed after the contact with any such reagents. Even an addition of concentrated solutions of NH_4_^+^, Mg^2+^, Ca^2+^, Ba^2+^, Li^+^, Na^+^, K^+^, and Zn^2+^ salts caused the formation of the starting materials **7**. The NMR spectra of dissolved **8** are typical for paramagnetic materials because only signals of the *n*-butyl groups of the counter ions were found and signals from the aliphatic periphery, whereas the signals of the aromatic core of radical anion were lost by strong line-broadening because of unfavorable relaxation processes. Spin and charge of radical anions **8** seem to be fully delocalised because both the carbonyl and C=C valence frequencies are lowered in the IR spectrum from 1688 and 1638 for the starting material [32] to 1601 and 1542 cm^−1^ for **8b** and even for the frequencies of the C=C vibrations from 1595 and 1579 to 1560 and 1492 cm^−1^ for **8b** indicating a weakening of these bonds. The radical-type character of the solids **8** is univocally indicated by their ESR spectra both of the solids and the re-dissolved material where signals with no hyper-fine structures were found; see below. The negative charge of **8** is indicated by anion mass spectrometry.

The more bathochromically shifted absorbing terrylenecarboximides [35] **1** (*n* = 3) were targeted as the next higher homologue; see Scheme 3. The formation of radical anions **2** (*n* = 3) was reported for the electrochemical reduction [36] of **1** (*n* = 3) and indicated by their UV/Vis/NIR absorption. Thus, we studied the isolation of **2** (*n* = 3) and found a minor pronounced tendency for hydrolysis compared with the lower homologues, presumably because of the higher electron density of **1** (*n* = 3). As a consequence, we substituted the nitrogen atoms of **1** (*n* = 3) with the highly solubilising 1-hexylheptyl group (swallow tail substituent) to prepare [37, 38] **10**. However, no **11** was obtained by a reduction with hydroxyacetone because a complete over reduction proceeded to the bis-anion **12** absorbing at 694 nm; see Fig. 4. The reducing aptitudes of hydroxyacetone and DBU could be balanced with the addition of diacetyl to the formation of the radical anion **11** indicated by a color change from deep blue to greenish blue. Finally, a precipitation with tetra-*n*-butylammonium bromide was successful to obtain the salt of **11** as a dark blue solid.

**Figure:**
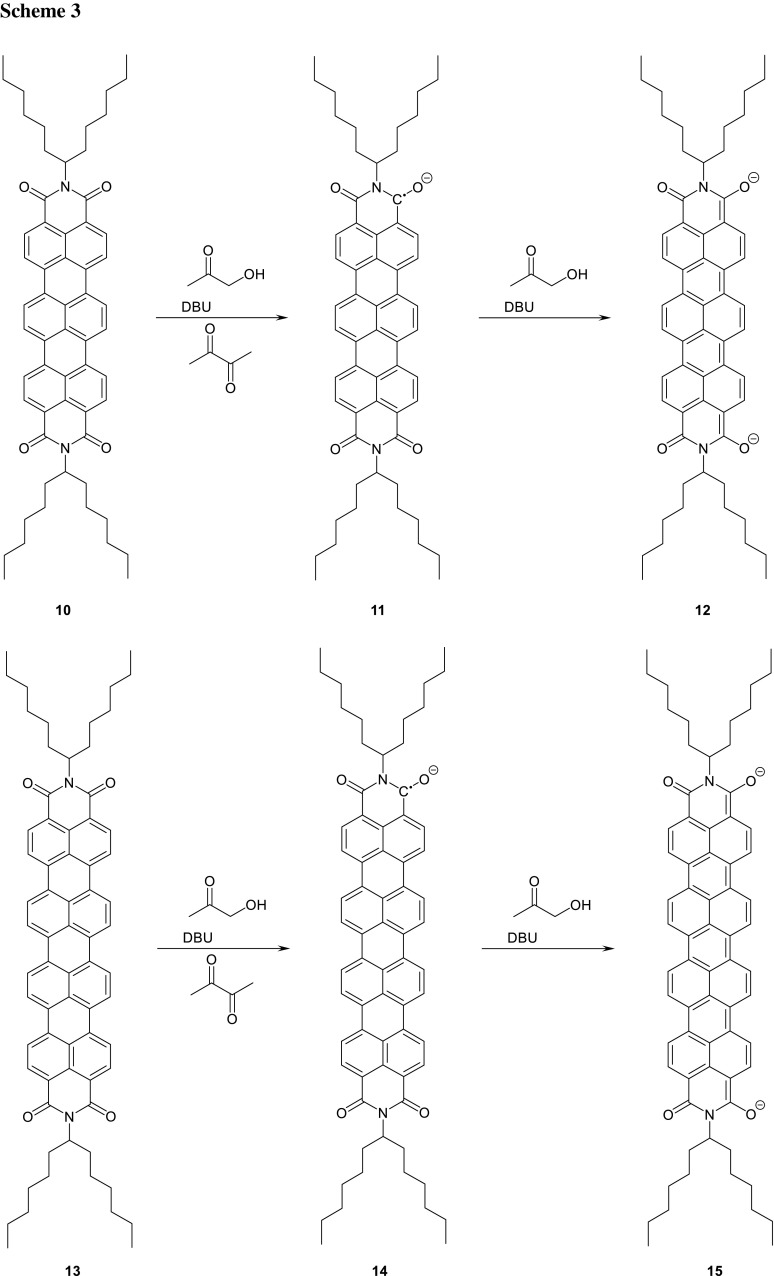

Quaterrylene tetracarboxybisimides [39, 40] **1** (*n* = 4) being known to be very bathochromically absorbing were rendered soluble [41, 42] by the *N*-(1-hexylheptyl) substituents to obtain **13** and reduced according to **4**. Complete over reduction proceeded to the slightly more bathochromically absorbing bis-anion **15** at 803 nm; see Fig. 5. The reducing aptitude of hydroxyacetone was controlled by the addition of diacetyl finally to obtain **14**. This radical anion could be precipitated by the addition of tetra-*n*-butylammonium bromide to obtain the radical salt of **14** as a dark green solid. The solid material can be stored under inert atmosphere without decomposition.

**Figure:**
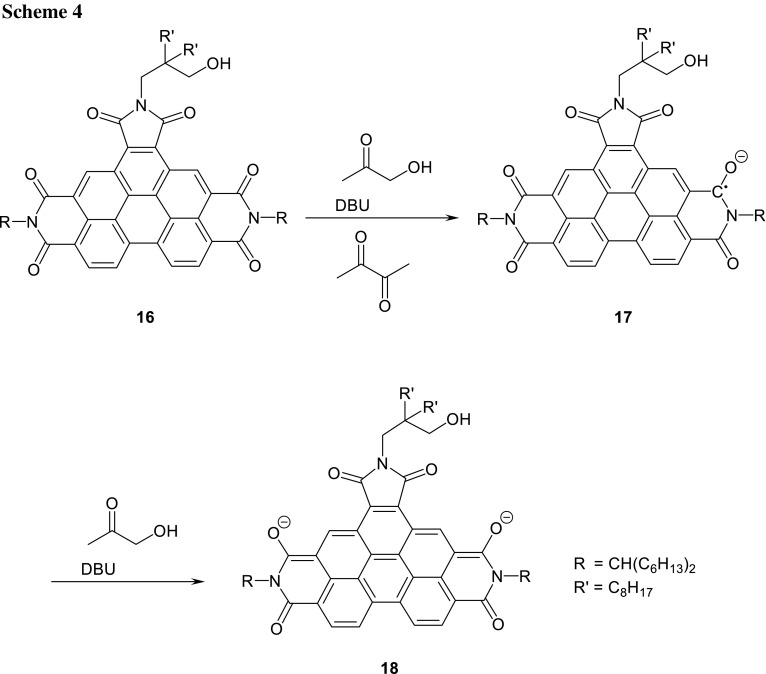

We extended the application of the method of reduction to even larger laterally conjugated systems. Thus, we studied the reduction of benzoperylenehexacarboxytrisimides [43] analogously to **1** to form **2** and tried to protect the starting material against alkaline hydrolysis analogously to **7** with 3-hydroxyalkyl groups. However, the synthesis of the staring material by the Clar variant of the Diels–Alder reaction of **7e** with maleic anhydride was not successful, presumably because of the still low solubility. As a consequence, we allowed to react the better soluble perylene derivative **1** (*n* = 3, R = 1-heyxlheptyl) to form the core-extended anhydride and tried to condense it with 2-aminomethyl-2-octyldecan-1-ol to form **16** to protect the more labile five-membered ring; Scheme 4. However, the condensation of the anhydride group with hydroxy amines by means of imidazole under standard conditions [44] was not successful, but could be achieved with DCC and TFA in chloroform [45] to form the benzoperylene hexacarboxytrisimide **16** with two efficiently solubilising [46] swallow-tail substituents (R) and one hydrolysis protecting group (R′) attached to the more labile five-membered ring causing an acceptable stabilization concerning hydrolysis. The benzoperylenehexacarboxytrisimide was reduced by the application of hydroxyacetone and precipitated with tetrabutylammonium bromide to form the salt of **17** as a yellowish dark green solid being much more sensitive versus oxygen than **8**. The precipitated **17** can be stored if oxygen is carefully excluded. The material is appreciably less stable in solution than **8** and rapidly decomposes during the UV/Vis spectroscopic measurements.

**Fig. 2:**
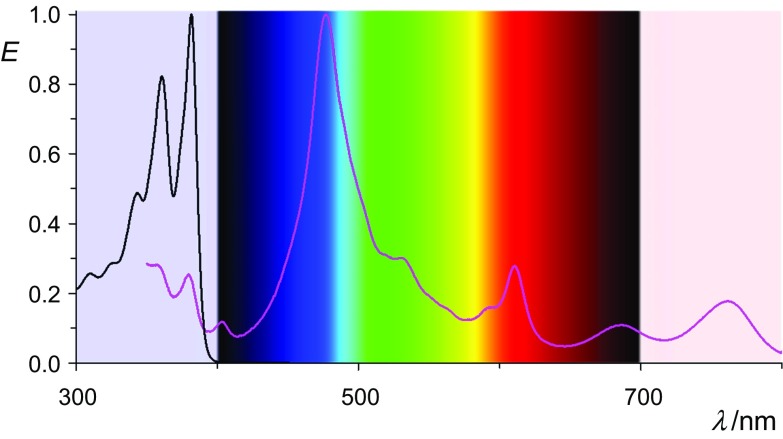
UV/Vis/NIR spectra of **5c** (in acetone, stabilized with hydroxyacetone, magenta curve) compared with **4c** (in chloroform, black curve in the UV)

The band-rich UV/Vis absorption spectrum of **5c** with the most bathochromic maximum at 761 nm is reaching NIR and is shown in Fig. 2. Fluorescence could not be detected as was expected.

**Fig. 3:**
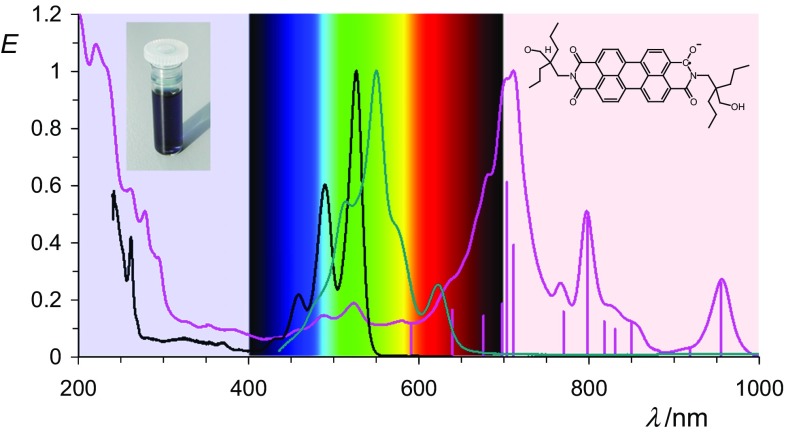
UV/Vis/NIR absorptions spectra of **8b** in acetonitrile (right line in magenta), the bis-anion **9b** (middle line in green) in acetone, and the starting material **7b** (left line in black) in chloroform. Bars: line positions obtained by a Gaussian analysis of the spectrum of **8b**

The solids of **8** form characteristically dark blue solutions in solvents such as acetonitrile or acetone where the color is not caused by the most bathochromic electron transition, but by a transition to higher electronic levels, and thus presents an example of colors of second order [47–50]. The most bathochromic absorption maximum is far in the NIR at 957 nm and there are many bands in the spectrum; see Fig. 3. The UV/Vis absorption of **8** corresponds to the previously reported absorption of the electrochemically [18, 29, 36] generated transient coloration by derivatives of **8**. Solid-state UV/Vis/NIR spectroscopy is used to confirm independently the radical character of **8** because a strong bathochromic absorption at 957 nm is observed being identical with respect to the dissolved material.

**Fig. 4:**
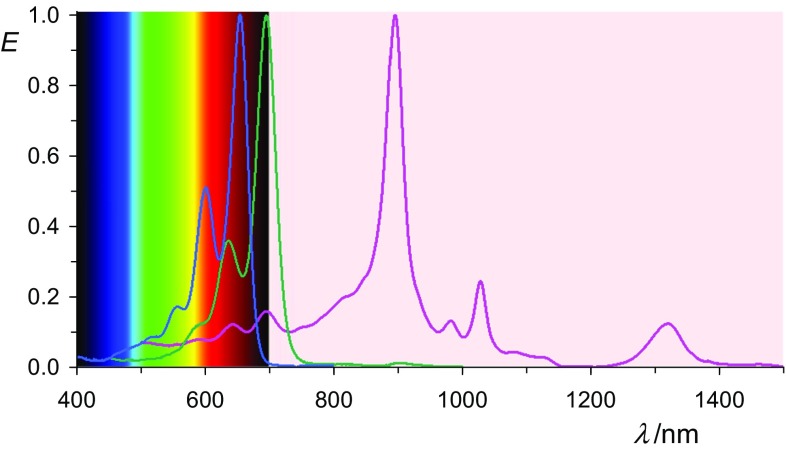
UV/Vis/NIR absorptions spectra of **11** stabilized in acetone (right line in magenta), the bis-anion **12** (middle in green) and the starting material **10** (left line in blue)

The terrylene-based radical anion **11** absorbs as bathochromic as 1320 nm; see Fig. 4. The absorption of the latter is mainly in the NIR, so that color is caused by residual bands in the visible.

**Fig. 5:**
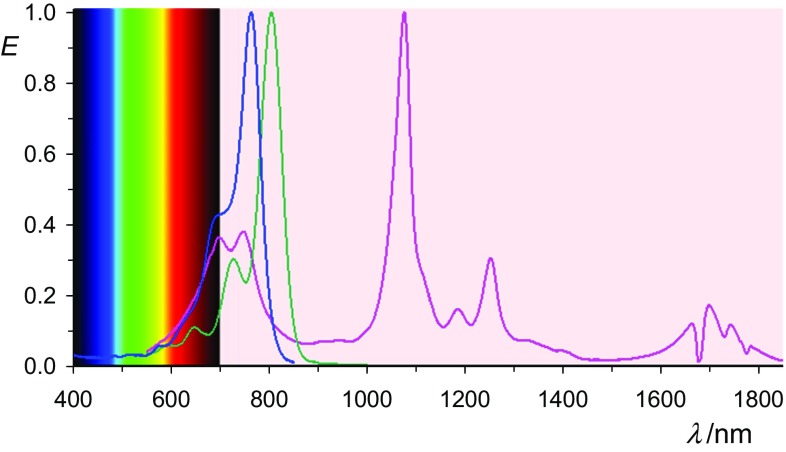
UV/Vis/NIR absorptions spectra of **14** stabilized in acetone (right line in magenta), the bis-anion **15** (middle line in green) and the starting material **13** (left line in blue); interference in the bathochromic region by the absorption of the medium

Finally, the quaterrylene-derived precipitated radical anion **14** was re-dissolved where the solution in acetone absorbs in the UV/Vis/NIR as bathochromic as 1699 nm being not far away from the region of vibration spectra; see Fig. 5.

**Fig. 6:**
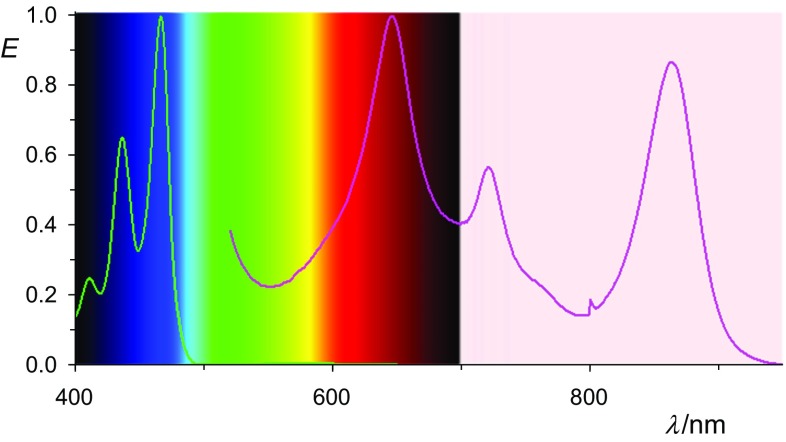
UV/Vis/NIR absorptions spectra of **17** stabilized in acetone (right line in magenta) with bathochromic-shifted feature with peak maxima at 718 nm and 867 nm and the starting material **16** (left line in green). Absorption maxima of the bis-anion **18** were found at 535 nm and 580 nm

The lateral extension of the perylenes to benzoperylenes causes a hypsochromic shift such as that shown in Fig. 6 for **16**. The UV/Vis spectrum of the radical anion **17** obtained by the reduction of **16** still extends to the NIR at 867 nm.

## Magnetic properties of the radical anions

**Fig. 7:**
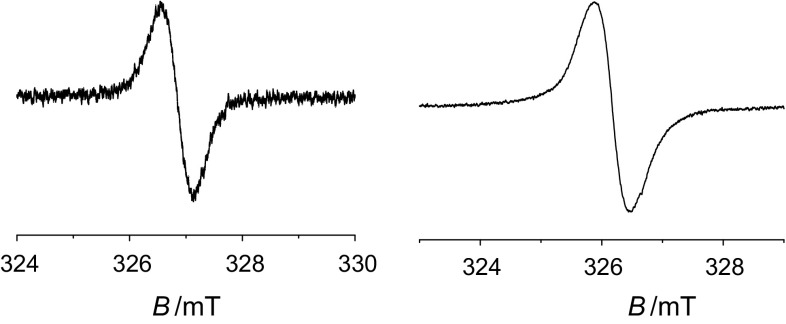
ESR spectra of **8a**. Left in acetone (*g* = 2.0038), right in the solid state (*g* = 2.0041)

The radical-type character of the solids **8** as well as in solutions is univocally indicated by their ESR spectra both of the solids and the re-dissolved material where signals with no hyper-fine structures were found; see Fig. 7.

### Magnetic measurements

**Fig. 8:**
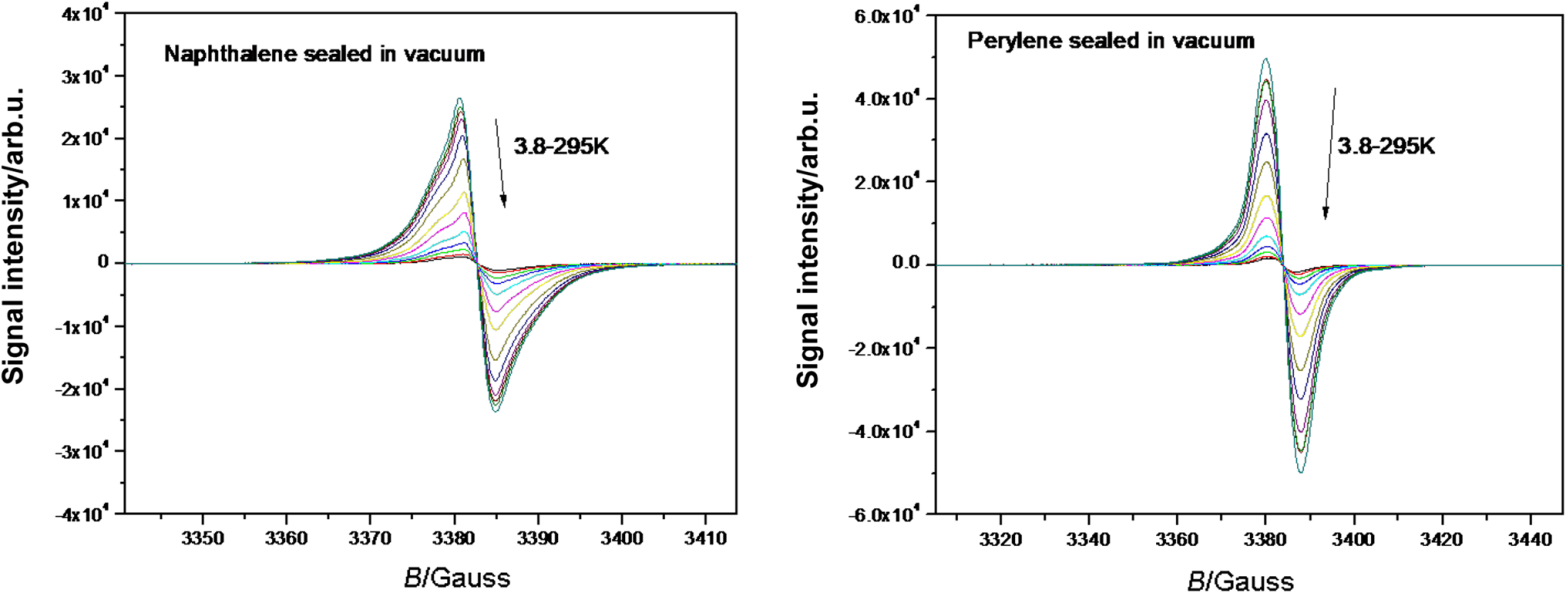
The temperature dependences of solid-state ESR signals recorded for the naphthalene (**2**, left) and the perylene (**4**, right) derivatives in the temperature range 3.8–300 K

Organic magnetic materials are of general interest because special effects can be expected such as the ferromagnetism, giant magnetoresistance, magnetic Seebeck effect, etc.; the low heat conductivity of organic materials is important for the latter [51–54]. The unpaired spin of **5a** and **8a**, respectively, persists in the solid state and allows the study of magnetic ordering. The ESR signal of the solid naphthalene derivative **5a** shows an irregular line shape which consists of more (up to three) overlapping lines with slightly shifted *g*-factors; Fig. 8, left. The *g*-factor of the main peak was found to be 2.0035. The signal undergoes line broadening upon cooling. We have observed change in amplitudes of individual signals after exposure to air which was partially reversible after re-evacuation. No detailed evaluation of signal amplitudes for samples exposed intentionally to air was performed due to the complex nature of their behavior.

The ESR signal of the solid perylene derivative **8a** shows a more regular line shape; Fig. 8, right. Unwanted passage effects led us to work at very low microwave power with attenuation set to 40 dB (20 µW), modulation amplitude 0.1 G, and modulation frequency 10 kHz to prevent any unwanted signal distortion. Perylene *g*-factor was found to be 2.0031. Small line broadening upon cooling was observed also in **7a** but not as significant as in the naphthalene derivative **4a**.

**Fig. 9:**
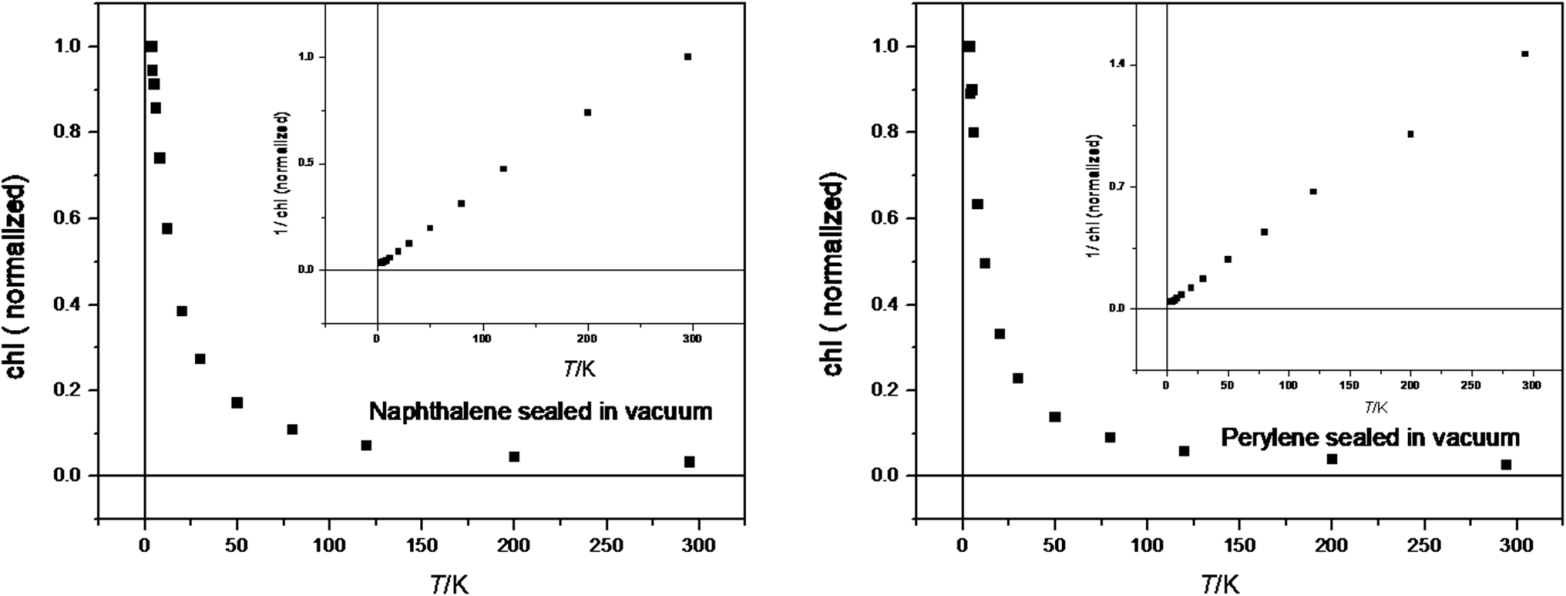
Temperature dependence of normalized magnetic susceptibility for solid naphthalene (**5a**, left) and perylene (**8a**, right) radicals anions evaluated from ESR measurements in the temperature range 3.8–300 K (squares). Plot of 1/*χ* vs. temperature is shown in the inserts

In the next step, we took a closer look at the magnetic behavior of the material. Figure 9 shows the normalized temperature dependence of doubly integrated ESR signals which corresponds to the magnetic susceptibility of the sample under study. Inset shows the temperature dependence of inverse susceptibility.

1 $$\chi \sim \frac{C}{{\left( {T - T_{C} } \right)^{\gamma } }}$$

**Fig. 10:**
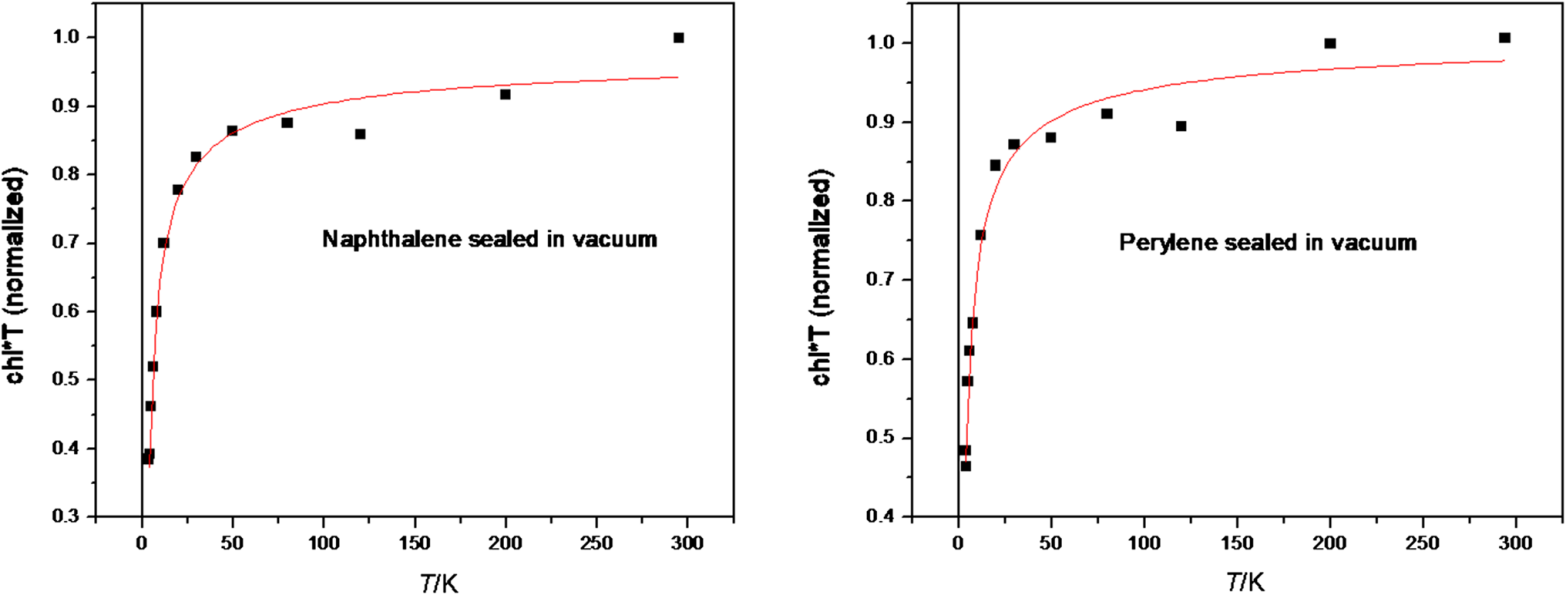
Temperature dependence of normalized magnetic susceptibility *T for naphthalene and perylene radicals evaluated from ESR measurements in the temperature range 3.8–300 K (squares). Continuous line is the best fit to Eq. (1)

Using the Curie–Weiss law (Eq. 1) where *C* is a Curie constant, *T* is the absolute temperature in Kelvin, *T*_*C*_ is the Curie temperature, and *γ* is critical exponent, it is possible to determine the character of magnetism (Fig. 10 fits). We observe that *T*_*C*_ and *γ* are close to 0 (i.e., 2–3 K) and close to unity, respectively. This indicates a paramagnetic behavior. Figure 10 shows in a more illustrative way the (paramagnetic) regime for both materials as the temperature dependence of the product *χ T*.

### Compressed solids

**Fig. 11:**
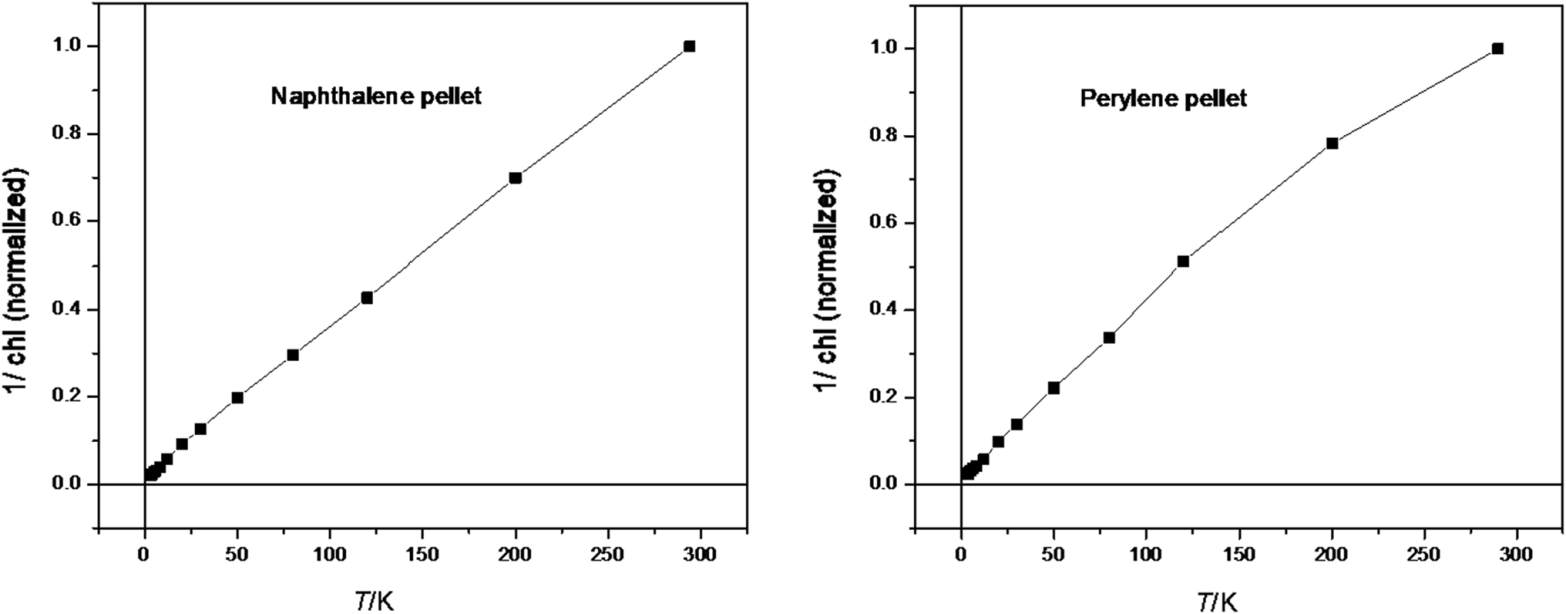
Temperature dependence of normalized magnetic susceptibility for compressed solid naphthalene (**4a**, left) and perylene (**7a**, right) radicals anions in the temperature range 3.8–300 K: plot of 1/*χ* vs. temperature

The solid materials **5a** and **8a** were compacted in vacuo by the application of pressure of 2700 bar (3 tons at 12 mm piston). The diagrams of Fig. 11 indicate paramagnetic properties for both compacted solids.

We calculated the number of free spins using the absolute intensities of the ESR signals. We found 1.5 × 10^16^ free spins at room temperature, where 1.5 × 10^16^ spins were found for 0.8 mg of compressed **5a** (1.9 × 10^19^ spins/g; 0.025 mol spins/mol) and 1.1 × 10^17^ for 2.8 mg of compressed **8a** (3.8 × 10^19^ spins/g; 0.060 mol spins/mol). This corresponds to one free spin for 40 molecules of **5a** and one free spin for 17 molecules of **8a**.

## Conclusion

**Fig. 12:**
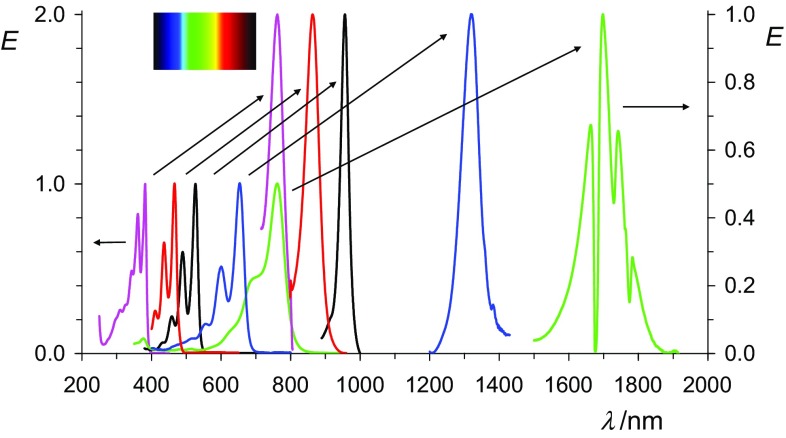
Overview: normalized UV/Vis/NIR absorptions spectra of oligo *peri*-arylenetetracarboxydiimides **1** in chloroform (lower lines, left with the left scale) compared with the most bathochromic-shifted absorption of their radical anions **2** in acetone (higher lines, right, right scale); from left to right: magenta: **4b**, red: **16**, black: **7b**, blue: **10**, green **13**; and magenta: **5b**, red: **17**, black: **8b**, blue: **11**, green **14**. Upper left: colors of the rainbow spectrum

We conclude that (bathochromically) shifted UV/Vis/NIR absorptions can be obtained by the reduction of suitable chromophores to their radical anions where an overview of *peri*-arylenes is shown in Fig. 12. The isolated radical anions promise many novel applications for dyestuff applications. The problems in conventional vat dying with the heavy-load of alkali being damaging delicate surfaces such as silk or human hair can be overcome by decoupling the processes of reduction and re-oxidation. The replacement of the environmentally problematic dithionite by hydroxyacetone or even electrochemical [55] processes for vat dyeing and the precipitation of the radical salts may turn such processes to green chemistry. The vat dying with the colorless **1** may be of special advantage because of sun-protection concerning UVA [56, 57]. This may be useful for the protecting of fibers and other materials, and also for sun-protecting clothes. Even the radical anions may be useful for many applications because of their bathochromic shifts of light absorption, their sensitivity against atmospheric oxygen can be solved by encapsulation such as in laminated compound glass.

## Experimental

All reagents were used as received from commercial suppliers. The solvents used in the reactions were dried with standard drying agents and freshly distilled prior to use. Reaction progress was monitored by thin-layer chromatography (TLC) on E. Merck Kieselgel 60 F254. Column chromatography was performed using silica gel (60 Å, 40–63 μm, ROCC). IR spectra were recorded as ATR with a Perkin Elmer 1420 Ratio Recording Infrared Spektrometer, FT 1000 (4000–450 cm^−1^). UV/Vis/NIR spectra: Varian Cary 5000; fluorescence spectra: Perkin Elmer FS 3000 (totally corrected). All ^1^H and ^13^C NMR spectra were recorded with a Varian Vnmrs 600 (600 MHz) in CDCl_3_ at 25 °C. Chemical shifts (*δ*) are reported in ppm and coupling constants (*J*) in Hz. Mass spectra were performed using a Finnigan MAT 95. Elemental analyses (C, H, N) were conducted using the Elemental Analyser Elementar Vario EL from Elementar Analysensysteme GmbH. ESR spectra were measured with an JES-RE2X ESR spectrometer from Jeol; X-band spectra in thin capillaries were recorded.

### 2-Cyano-2-octyldecanoic acid methyl ester (C_20_H_37_NO_2_)

18 cm^3^ cyanoacetic acid methyl ester (200 mmol) and 70 cm^3^ 1-bromooctane (400 mmol) were slightly warmed with the exclusion of air and treated dropwise with 91 cm^3^ of a solution of 25% sodium methanolate in methanol (400 mmol), refluxed for 8 h, stirred at room temperature for 16 h, treated with 100 cm^3^ distilled water for the dissolution of the precipitate of sodium bromide, concentrated by the evaporation of methanol, collected by the extraction with 1 × 100 cm^3^ and 2 × 50 cm^3^ diethyl ether, dried with magnesium sulfate, evaporated, and distilled in medium vacuo. Yield 43.5 g (135 mmol, 67%) colorless liquid; b.p.: 155–162 °C (9 × 10^−3^–1.6 × 10^−2^ mbar); IR (ATR): $\bar{\nu}$ = 2955 (s), 2925 (s), 2856 (s), 2244 (w), 1745 (s), 1458 (m), 1379 (w), 1232 (m), 1177 (w), 1137 (w), 1079 (w), 775 (w), 723 cm^−1^ (w); ^1^H NMR (600 MHz, CDCl_3_): *δ* = 0.88 (t, 6H, ^*3*^*J *= 7.0 Hz, –CH_3_) 1.30 (m, 22H, –CH_2_), 1.55 (m, 2H, –CH_2_), 1.76 (m, 2H, –CH_2_), 1.88 (dt, 2H, ^*3*^*J *= 4.0 Hz, ^*2*^*J *= 13.3 Hz, CH_2_), 3.81 (s, 3H, –OCH_3_) ppm; ^13^C NMR (150 MHz, CDCl_3_): *δ* = 14.1, 22.6, 25.4, 29.1, 29.2, 31.8, 50.0, 53.2, 119.5, 169.9 ppm; MS (DEI^+^/70 eV): *m/z* (%) = 324 (1), 323 (3) [M^+^], 322 (3), 264 (15) [M^+^-CO_2_CH_3_], 212 (7), 211 (42) [M^+^-C_8_H_17_], 210 (6), 156 (9), 155 (100) [M^+^-C_8_H_17_, –CO_2_CH_3_), 154 (14), 39 (18); HRMS: *m/z* calcd. 323.2824 (C_20_H_37_NO_2_), found 323.2836, Δ = 1.2 mmu.

### 2-Aminomethyl-2-octyldecan-1-ol (C_19_H_41_NO)

5.92 g LiAlH_4_ (156 mmol, 1.3 equiv.) was disperged under argon in 100 cm^3^ anhydrous *tert*-butyl methyl ether, diluted with 50 cm^3^*tert*-butyl methyl ether, treated dropwise with stirring and ice cooling with 38.8 g 2-cyano-2-octyldecanoic acid methylester (120 mmol) in 50 cm^3^*tert*-butyl methyl ether, treated with further 400 mg LiAlH_4_ (11 mmol), refluxed for 1 h, stirred at room temperature for 16 h, treated cautiously dropwise and with vigorous stirring and ice cooling with 6 cm^3^ distilled water, 6 cm^3^ 2 n aqueous NaOH, and 20 cm^3^ distilled water, stirred for 2 h and filtered. The finely powdered solid was three times refluxed with 50 cm^3^ each *tert*-butyl methyl ether (30 min) and discarded. The combined organic phases were washed with distilled water, dried with magnesium sulfate, and distilled with Kugelrohr in medium vacuum. Yield 24.5 g (81.7 mmol, 68%) colorless liquid; b.p.: 205–225 °C (1 × 10^−3^–2 × 10^−3^ mbar); IR (ATR): $\bar{\nu}$ = 3368 (w, br), 3300 (w, br), 1596 (w), 1466 (m), 1378 (w), 1051 (m), 721 (w) cm^−1^; ^1^H NMR (600 MHz, CDCl_3_): *δ *= 0.88 (t, 6H, ^*3*^*J* = 7.0 Hz, –CH_3_), 1.26 (m, 28H, –CH_2_), 2.77 (s, 2H, –C*H*_2_–NH_2_), 3.56 (s, 2H, –C*H*_2_–OH) ppm; ^13^C NMR (150 MHz, CDCl_3_): *δ *= 14.1, 22.7, 23.0, 29.3, 29.6, 30.6, 31.9, 50.1, 71.5 ppm.

### 2,7-Bis(2-ethyl-2-hydroxymethylbutyl)benzo[*lmn*][3,8]phenanthroline-1,3,6,8-tetraone (**4a**, C_28_H_34_N_2_O_6_)

410 mg Isochromeno[6,5,4-*def*]isochromene-1,6,8-tetraone (1.53 mmol), 600 mg 2-aminomethyl-2-ethylbutane-1-ol (4.60 mmol), and 25 cm^3^ DMF under nitrogen were refluxed at 100 °C for 2 h (color change to red), allowed to cool, treated cautiously with ice cooling with 50 cm^3^ 2 n aqueous HCl, collected by vacuum filtration, washed with a small amount of distilled water, dried at 80 °C in air, dispersed in a small amount of methanol, refluxed with 10% aqueous K_2_CO_3_, collected by vacuum filtration, washed with methanol/water 1:1, and dried at 80 °C in air. Yield 202 mg (26%) slightly rose solid; m.p.: 255–258 °C; IR (ATR): $\bar{\nu}$ = 3550 (s), 3508 (m), 2967 (s), 2883 (m), 1702 (s), 1657 (s), 1580 (m), 1549 (w), 1454 (m), 1428 (w), 1372 (m), 1329 (s), 1247 (m), 1168 (w), 1105 (w), 1030 (w), 1001 (w), 889 (w), 772 (m) cm^−1^; ^1^H NMR (CDCl_3_, 600 MHz): *δ *= 0.94 (t, 12H, ^3^*J* = 7.5 Hz, –CH_3_), 1.32 (qd, 4H, ^*3*^*J* = 7.5 Hz, ^*2*^*J *= 14.7 Hz, –CH_2_–), 1.41 (qd, 4H, ^*3*^*J* = 7.5 Hz, ^*2*^*J *= 14.9 Hz, –CH_2_–), 3.23 (d, 4H, ^3^*J* = 7.6 Hz, –CH_2_–OH), 3.62 (t, 2H, ^3^*J* = 7.6 Hz, –CH_2_–O*H*), 8.79 (s, 4H, CH_ar_) ppm; ^13^C NMR (CDCl_3_, 600 MHz): *δ *= 7.4, 23.7, 43.1, 43.7, 65.2, 126.5, 126.6, 131.6, 164.3 ppm; UV/Vis (CHCl_3_): *λ*_max_ = 383, 362, 344 nm; MS (ESI): *m/z* (%) = 641 (82) [M^+^+^35^Cl], 605 (47) [M^+^−H^+^]; HRMS/ESI: *m/z* calcd. 494.2417 (C_28_H_34_N_2_O_6_), found 494.2429, Δ = 1.2 mmu.

### 2,7-Bis(2-hydroxymethyl-2-propylpentyl)benzo[*lmn*][3,8]phenanthroline-1,3,6,8-tetraone (**4b**, C_32_H_42_N_2_O_6_)

655 mg Isochromeno[6,5,4-*def*]isochromene-1,3,6,8-tetraone (2.45 mmol), 1.17 g 2-aminomethyl-2-propylpentane-1-ol (7.34 mmol), and 75 cm^3^ DMF were allowed to react and purified analogously to 2,7-bis(2-ethyl-2-hydroxymethylbutyl)benzo[*lmn*][3,8]phenanthroline-1,3,6,8-tetraone (**4a**). Yield 750 mg (56%) slightly rose solid; m.p.: 199–201 °C; IR (ATR): $\bar{\nu}$ = 3478 (s), 2953 (s), 2928 (m), 2871 (m), 1698 (s), 1642 (s), 1582 (m), 1458 (m), 1433 (m), 1374 (m), 1330 (s), 1244 (m), 1202 (m), 1161 (w), 1094 (m), 1039 (w), 1016 (m), 975 (w), 890 (w), 860 (w), 776 (m), 720 (w), 662 (w) cm^−1^; ^1^H NMR (300 MHz, CDCl_3_): *δ *= 0.94 (t, 12H, ^3^*J* = 7.3 Hz, –CH_3_), 1.34 (m, 12H, –CH_2_), 1.50 (m, 4H, –CH_2_), 3.20 (d, 4H, ^3^*J* = 7.6 Hz –C*H*_2_–OH), 3.70 (t, 2H, ^3^*J* = 7.6 Hz, –CH_2_–O*H*), 4.20 (s, 4H, –C*H*_2_–NR_2_), 8.79 (s, 4H, CH_ar_) ppm; ^13^C NMR(600 MHz, CDCl_3_): *δ* = 14.96, 16.28, 34.38, 43.25, 43.99, 65.68, 126.5, 131.6, 164.3 ppm; UV/Vis (CHCl_3_): *λ*_max_ (*ε*) = 383 (26,600), 362 (22,000), 344 (13,400) nm (dm^3^ mol^−1^ cm^−1^); MS (DEI^+^, 70 eV): *m/z* (%) = 550 (15) [M^+^], 520 (100) [M^+^−CH_3_O], 422 (7), 410 (12), 409 (53), 294 (9), 281 (13), 268 (6).

### 2,7-Bis(2-butyl-2-hydroxymethylhexyl)benzo[*lmn*][3,8]phenanthrolin-1,3,6,8-tetraone (**4c**, C_40_H_42_N_2_O_6_)

1.31 g Isochromeno[6,5,4-*def*]isochromene-1,3,6,8-tetraone (4.89 mmol), 2.75 g 2-aminomethyl-2-butylhexane-1-ol (14.7 mmol), and 70 cm^3^ DMF were allowed to react and purified analogously to 2,7-bis(2-ethyl-2-hydroxymethylbutyl)benzo[*lmn*][3,8]phenanthroline-1,3,6,8-tetraone (**4a**). Yield 985 mg (1.62 mmol, 33%) colorless, shiny solid; m.p.: 232–234 °C; IR (ATR): $\bar{\nu}$ = 3480 (s), 2953 (s), 2930 (s), 2864 (m), 1698 (s), 1646 (s), 1581 (m), 1459 (m), 1432 (m), 1367 (m), 1330 (s), 1244 (s), 1199 (w), 1150 (w), 1099 (w), 1024 (m), 884 (w), 865 (w), 776 (w), 720 (w), 660 (w) cm^−1^; ^1^H NMR (CDCl_3_, 600 MHz): *δ* = 0.92 (t, 12H, ^*3*^*J* = 7.1 Hz, –CH_3_), 1.22–1.33 (m, 20H, –CH_2_–), 1.38–1.46 (m, 4H, –CH_2_–), 3.21 (d, 4H, ^*3*^*J* = 7.6 Hz, –C*H*_2_–OH), 3.67 (t, 2H, ^*3*^*J* = 7.6 Hz, –CH_2_–O*H*), 4.21 (s, 4H, –C*H*_2_–NR_2_), 8.79 (s, 4H, CH_ar_) ppm; ^13^C NMR (CDCl_3_, 600 MHz): *δ* = 14.07, 23.60, 25.1, 31.65, 43.02, 43.98, 65.70, 126.51, 131.58, 164.25 ppm; MS (ESI): *m/z* (%) = 641 (82) [M^+^+^35^Cl], 605 (47) [M^+^−H]; UV/Vis (CHCl_3_): *λ*_max_ (*ε*) = 383 (26,600), 362 (21,900), 344 (13,400) nm (dm^3^ mol^−1^ cm^−1^).

### Bis(2-butyl-2-hydroxymethylhexyl)benzo[*lmn*][3,8]phenanthroline-1,3,6,8-tetraone (**4d**, C_40_H_58_N_2_O_6_)

1.31 g Isochromeno[6,5,4-*def*]isochromene-1,3,6,8-tetraone **13** (4.89 mmol), 3.16 g 2-aminomethyl-2-pentylheptan-1-ol (14.7 mmol), and 75 cm^3^ DMF were allowed to react and purified analogously to 2,7-bis(2-ethyl-2-hydroxymethylbutyl)benzo[*lmn*][3,8]phenanthroline-1,3,6,8-tetraone (**4a**). Yield 575 mg (867 µmol, 18%) colorless, shiny solid; m.p.: 226–229 °C; IR (ATR): $\bar{\nu}$ = 3486 (s), 2930 (s), 2862 (m), 1698 (s), 1644 (s), 1581 (m), 1460 (m), 1431 (w), 1413 (w), 1369 (w), 1330 (s), 1242 (m), 1209 (w), 1191 (w), 1150 (w), 1104 (w), 1058 (w), 1026 (m), 890 (w) 863 (vw), 778 (w) 719 (w) 662 (w) cm^−1^; ^1^H NMR (CDCl_3_, 600 MHz): *δ *= 0.92 (t, 12H, ^3^*J* = 7.2 Hz, –CH_3_), 1.19–1.37 (m, 28H, –CH_2_–), 1.38–1.48 (m, 4H, –CH_2_–), 3.20 (d, 4H, ^3^*J* = 7.6 Hz, –C*H*_2_–OH), 3.65 (t, 2H, ^3^*J* = 7.6 Hz, –CH_2_–O*H*), 8.79 (s, 4H, CH_ar_) ppm; ^13^C NMR (CDCl_3_, 600 MHz): *δ *= 14.12, 22.59, 31.95, 32.79, 43,10, 65.72, 126.50, 131.57, 164.24 ppm; UV/Vis (CHCl_3_): *λ*_max_ (*ε*) = 383 (26,600), 362 (22,000), 344 (13,800) nm (dm^3^ mol^−1^ cm^−1^); MS (ESI): *m/z* (%) = 697 (68) [M^+^+^35^Cl], 661 (47) [M^+^−H].

### 2,7-Bis(2-hydroxymethyl-2-octyldecyl)benzo[*lmn*][3,8]phenanthroline-1,3,6,8-tetraone (**4e**, C_52_H_81_N_2_O_5_)

1.50 g Isochromeno[6,5,4-*def*]isochromene-1,3,6,8-tetraone (5.59 mmol), 5.30 g 2-aminomethyl-2-octyldecane-1-ol (16.8 mmol), and 170 cm^3^ DMF were allowed to react and purified analogously to 2,7-bis(2-ethyl-2-hydroxymethylbutyl)benzo[*lmn*][3,8]phenanthroline-1,3,6,8-tetraone (**4a**) and recrystallised two times from chloroform and purified by column separation (silica gel 60, chloroform/acetic acid 50:1). Yield 1.74 g (2.09 mmol, 37%) slightly rose, shiny solid; m.p.: 176–178 °C; *R*_*f*_ = 0.5 (silica gel, cloroform/acetic acid 50:1); IR (ATR): $\bar{\nu}$ = 3481 (s), 2956 (m), 2921 (s), 2852 (s), 1699 (s), 1645 (s), 1580 (s), 1460 (s), 1432 (w), 1415 (w), 1387 (w), 1370 (w), 1331 (s), 1255 (w), 1245 (m), 1191 (w), 1152 (w), 1112 (w), 1094 (w), 1027 (w), 975 (w), 891 (w), 865 (w), 815 (w), 779 (w), 720 (w) cm^−1^; ^1^H NMR (600 MHz, CDCl_3_): *δ *= 0.88 (t, 12H, ^*3*^*J* = 7.0 Hz, –CH_3_), 1.20–1.33 (m, 50H, –CH_2_–), 1.36–1.45 (m, 4H, –CH_2_–), 1.54–1.60 (m, 2H, –CH_2_–), 3.20 (s, 4H, –C*H*_2_–NR_2_), 3.66 (s, br, 2H, –OH), 8.79 (s, 4H, CH_ar_) ppm; ^13^C NMR (600 MHz, CDCl_3_): *δ* = 14.11, 22.66, 22.89, 29.31, 29.51, 29.53, 30.55, 30.57, 31.87, 31.95, 31.98, 43.08, 43.97, 65.69, 126.48, 131.56, 164.21, 164.23 ppm; UV/Vis (CHCl_3_): *λ*_max_ (*ε*) = 383 (26,900), 362 (22,300), 344 (14,000) nm (dm^3^ mol^−1^ cm^−1^); MS (ESI): *m/z* (%) = 865.6 (100) [M^+^+^35^Cl], 830.6 (44) [M^+^−H]; HRMS: *m/z* calcd. 831.6173 (C_52_H_81_N_2_O_5_), found 831.6290, Δ = 1.1 mmu.

### *N*,*N*′-Bis(1-hexylheptyl)quaterrylene-3,4,13,14-tetracarboxylic acid 3,4:13,14-diimide (**13**, C_70_H_70_N_2_O_4_) [31]

200 mg 9,9′-Bis[*N*-(1-hexylheptyl)perylene-3,4-dicarboximide] (196 mmol) and 1.26 g potassium carbonate (9.31 mmol) were suspended in 1.8 cm^3^ ethanolamine heated with stirring at 160 °C for 4 h, allowed to cool, treated with 10 cm^3^ methanol, collected by vacuum filtration, washed with plenty of water, dried at 100 °C in air for 16 h, purified by column separation (silica gel, dichloromethane), dissolved in a small amount of dichloromethane, precipitated with plenty of methanol, and dried at 100 °C in air for 16 h. Yield 96 mg (48%) blue solid; m.p.: > 250 °C; *R*_*f*_ = 0.07 (silica gel/CH_2_Cl_2_); IR (ATR): $\bar{\nu}$ = 2920 (s), 2851 (s), 1688 (s), 1645 (s), 1595 (m), 1571 (s), 1502 (m), 1456 1404 (m), 1372 (w), 1344 (s), 1284 (m) 1219 (w), 1171 (w), 1104 (w), 1047 (w), 836 (w) 804 (m), 745 (m), 669 (w) cm^−1^; ^1^H NMR (CDCl_3_, 600 MHz): *δ *= 0.85–0.95 (t, br, 12H –CH_3_), 1.14 (m, 32H, –CH_2_), 2.00 (m, br, 4H, R_2_NCH–C*H*_2_–R), 2.30 (m, 4H, R_2_NCH–C*H*_2_–R), 5.19 (m, 1H, R_2_NC*H*–), 7.72–7.85 (m, 12H, CH_ar_), 8.23 (s, 4H, CH_ar_) ppm; ^13^C NMR (CDCl_3_, 150 MHz): *δ *=14.4, 2.9, 27.5, 29.6, 29.9, 31.2, 32.1, 32.7, 54.9, 120.4, 122.4, 123.8, 125.8, 127.3, 127.9, 129.2, 129.9, 131.3, 135.3, 164.8 ppm; UV/Vis: *λ*_max_ (*E*_*rel*_) = 762 (1.00), 694 (sh., 0.18), 377 nm (sh., 0.09); MS (ESI): *m/z* = 1002.5 [M^+^].

### *N*,*N*″-Bis(1-hexylheptyl)-*N*′-(2-hydroxymethyl-2-octyldecyl)benzoperylene-1′,2′,3,4,9,10-hexacarboxylic acid 1′,2′:3,4:9,10-trisimide (**16**, C_73_H_99_N_3_O_7_)

500 mg *N*,*N′*-Bis(1-hexylheptyl)benzo[*g,h,i*]perylene-2,3,8,9,11,12-hexacarboxylic acid 2,3;8,9-bisimide-11,12-anhydride (590 μmol), 609 mg dicyclohexyl carbodiimide (295 μmol), and 931 mg 2-aminomethyl-2-octyldecane-1-ol (295 μmol) were dissolved in 18 cm^3^ chloroform, treated with three drops of trifluoroacetic acid, refluxed for 18 h, allowed to cool, treated with 80 cm^3^ distilled water, stirred at room temperature (3 h), diluted with 350 cm^3^ chloroform, extracted three times with 300 cm^3^ each 2 n aqueous HCl, dried over magnesium sulfate, evaporated, dissolved in a small amount of chloroform, precipitated with plenty of methanol, collected by vacuum filtration, purified by column separation (silica gel, toluene to remove the starting material) and a second column separation (silica gel, isohexane to remove aliphatic by-product and elution with toluene for the main fraction), dissolved in a small amount of chloroform and precipitated with methanol, collected by vacuum filtration, and dried at 80 °C in air. Yield 137 mg (21%) yellow solid; m.p.: 289–292 °C; *R*_*f*_ = 0.5 (toluene); IR (ATR): $\bar{\nu}$ = 3551 (m), 2955 (s), 2924 (s), 2855 (s), 1766 (w), 1707 (m), 1663 (m), 1625 (w), 1596 (w), 1523 (w), 1457 (w), 1414 (w), 1397 (w), 1364 (w), 1316 (m), 1239 (w), 1175 (w), 1103 (w), 945 (w), 845 (w), 812 (w), 764 (w), 747 (w) cm^−1^; ^1^H NMR (600 MHz, CDCl_3_): *δ* = 0.84 (t, 12H, ^*3*^*J* = 7.0 Hz, –CH_3_), 0.89 (t, 6H, ^*3*^*J* = 6.9 Hz, –CH_3_), 1.23–1.58 (m, 56H, –CH_2_), 2.01 (m, br, 4H, NR_2_–CH–C*H*_2_), 2.38 (m, br, 4H, NR_2_–CH–C*H*_2_), 3.39 (s, 2H, –C*H*_2_–OH), 3.73 (s, br, 1H, –CH_2_–O*H*), 3.93 (s, 2H, –C*H*_2_–NR_2_), 9.10 (s, br, 2H, CH_ar_), 9.18 (s, br, 2H, CH_ar_), 10.22 (s, br, 2H, CH_ar_) ppm; ^13^C NMR (150 MHz, CDCl_3_): *δ* = 14.0, 22.7, 27.1, 29.3, 29.4, 29.7, 30.6, 31.8, 31.9, 42.9, 55.4, 65.5, 122.9, 123.8, 124.4, 127.0, 127.4, 132.8, 169.6 ppm; UV/Vis (CHCl_3_): *λ*_max_ (*ε*)= 379 (43,000), 411 (16,000), 436 (39,000), 466 (61,000) nm (dm^3^ mol^−1^ cm^−1^); Fluorescence (CHCl_3_): *λ*_max_ = 475, 509 nm; Fluorescence quantum yield (CHCl_3_, *E* = 0.0338 cm^−1^, *λ*_*exc*_ = 435 nm, reference perylene-3,4,9,10-tetracarboxylic acid tetramethyl ester with *Φ* = 100%): 32%; MS (DEI^+^, 70 eV): *m/z* (%) = 1132.1 (29), 1131.1 (29), 1130.1 (64), 1129.1 (83) [M^+^], 1116.1, 1099.1 (46), 950.1 (13), 949.1 (37), 948.1 (61), 917.1 (46), 861.1 (27), 860.1 (21), 737.1 (46), 735.1 (38), 680.0 (49), 679.0 (81), 678.0 (42), 666.0 (25), 509.9 (11), 499.0 (19), 498.0 (58), 496.9 (100), 495.9 (74), 483.9 (58), 482.9 (34), 414.0 (18), 111.2 (17), 83.2 (35), 69.2 (47); HRMS/ESI: *m/z* calcd. 1131.7640 (C_73_H_101_N_3_O_7_), found 1131.7643, Δ = 0.3 mmu.

### 2,7-Bis(2-ethyl-2-hydroxymethylbutyl)-3,6,8-trioxo-1,2,3,6,7,8-hexahydrobenzo[*lmn*][3,8]phenanthrolin-1-ol radical anion tetrabutylammonium salt (**5a**, C_28_H_34_N_2_O_6_·C_16_H_36_N)

122 mg 2,7-Bis(2-ethyl-2-hydroxymethylbutyl)benzo[*lmn*][3,8]phenanthroline-1,3,6,8-tetraone (**4a**, 240 μmol) under argon was dispersed in 0.5 cm^3^ degassed ethanol, treated with 1 cm^3^ degassed distilled water and 1 cm^3^ DBU, heated at 45 °C, treated with 1 cm^3^ hydroxyacetone (15 mmol), stirred for 10 min, treated with 1.0 g tetrabutylammonium bromide (3.1 mmol) in 1.5 g degassed distilled water, diluted with 10 cm^3^ degassed distilled water, stirred at 0 °C for 10 min, collected by vacuum filtration under argon, washed with degassed distilled water until colorless washings, dried in medium vacuum and then in a countercurrent of nitrogen over phosphorous pentoxide. Yield 38 mg (21%) brownish black solid; m.p.: > 300 °C; IR (ATR): $\bar{\nu}$ = 3332 (m, br), 2959 (s), 2875 (m), 1632 (s), 1580 (s), 1558 (s), 1520 (s), 1458 (m), 1376 (m), 1298 (m), 1146 (w), 1068 (m), 876 (w), 798 (w), 756 (w) cm^−1^; UV/Vis (acetone): *λ*_max_ (*E*_*rel*_) = 477 (1.00), 611 (0.24), 686 (0.06), 761 (0.14) nm.

### 2,7-Bis(2-hydroxymethyl-2-propylpentyl)-3,6,8-trioxo-1,2,3,6,7,8-hexahydrobenzo[*lmn*][3,8]phenanthrolin-1-ol radical anion tetrabutylammonium salt (**5b**, C_32_H_42_N_2_O_6_·C_16_H_36_N)

132 mg 2,7-Bis(2-hydroxymethyl-2-propylpentyl)benzo[*lmn*][3,8]phenanthroline-1,3,6,8-tetraone (**4b**, 0.24 mmol) was allowed to react and purified as was described for 2,7-bis(2-ethyl-2-hydroxymethylbutyl)-3,6,8-trioxo-1,2,3,6,7,8-hexahydrobenzo[*lmn*][3,8]phenanthrolin-1-ol radical anion tetrabutylammonium salt (**5a**). Yield 97 mg (0.11 mmol, 48%) brownish black solid; m.p.: > 300 °C; IR (ATR): $\bar{\nu}$ = 3317 (m, br), 2958 (s), 2931 (m), 2872 (s), 1621 (s), 1577 (s), 1555 (m), 1516 (s), 1488 (w), 1461 (m), 1435 (w), 1379 (w), 1324 (m), 1296 (m), 1232 (w), 1204 (w), 1150 (w), 1103 (w), 1053 (w), 1010 (w), 880 (w), 853 (w), 812 (w), 744 (w) cm^−1^; ^1^H NMR (400 MHz, acetone-*d*_*6*_): *δ* = 0.97 (t, 12H, ^3^*J* = 7 Hz, R_3_N–(CH_2_)_3_–C*H*_3_), 1.42 (m, 8H, R_3_N–(CH_2_)_2_–C*H*_2_–CH_3_), 1.80 (q, br, 8H, R_3_N–CH_2_–C*H*_2_–CH_2_–CH_3_), 3.42 (t, 8H, ^3^*J* = 8 Hz, R_3_N–C*H*_2_–(CH_2_)_2_–CH_3_) ppm; ESR (solid state): *g *= 2.0043; ESR (acetone): *g *= 2.0042; UV/Vis (acetone): *λ*_max_ (*E*_*rel*_) = 477 (1.00), 611 (0.13), 686 (0.08), 761 (0.02) nm; MS (FAB^−^): *m/z* (%) = 550.3 (100) [M^−^ (C_32_H_42_N_2_O_6_^−^)]; HRMS: *m/z* calcd. 550.3048 (C_32_H_42_N_2_O_6_^−^), found 550.3063, Δ = 1.5 mmu.

### 2,7-Bis(2-butyl-2-hydroxymethylhexyl)-3,6,8-trioxo-1,2,3,6,7,8-hexahydrobenzo[*lmn*][3,8]phenanthrolin-1-ol radical anion tetrabutylammonium salt (**5c**, C_40_H_42_N_2_O_6_·C_16_H_36_N)

146 mg 2,7-Bis(2-butyl-2-hydroxymethylhexyl)benzo[*lmn*][3,8]phenanthroline-1,3,6,8-tetraone (**4c**, 0.24 mmol) was allowed to react and purified as was described for 2,7-bis(2-ethyl-2-hydroxymethylbutyl)-3,6,8-trioxo-1,2,3,6,7,8-hexahydrobenzo[*lmn*][3,8]phenanthrolin-1-ol radical anion tetrabutylammonium salt (**5a**). Yield 117 mg (58%) brownish black powder; m.p.: > 300 °C; IR (ATR): $\bar{\nu}$ = 3300 (m, br), 3110 (m, br), 2956 (s), 2929 (s), 2872 (s), 1621 (s), 1587 (w), 1572 (w), 1556 (m), 1523 (s), 1493 (w), 1463 (m), 1424 (w), 1400 (w), 1380 (w), 1364 (w), 1293 (m), 1196 (w), 1145 (w), 1106 (w), 1066 (w), 1053 (w), 1026 (w), 984 (w), 935 (w), 879 (w), 815 (w), 743 (w) cm^−1^; ^1^H NMR (400 MHz, acetone-*d*_*6*_): *δ* = 0.98 (t, 12H, ^3^*J* = 7 Hz, R_3_N–(CH_2_)_3_–C*H*_3_), 1.43 (m, 8H, R_3_N–(CH_2_)_2_–C*H*_2_–CH_3_), 1.82 (q, br, 8H, R_3_N–CH_2_–C*H*_2_–CH_2_–CH_3_), 3.46 (t, 8H, ^3^*J* = 8 Hz, R_3_N–C*H*_2_–(CH_2_)_2_–CH_3_) ppm; ESR (acetone): *g *= 2.0039; UV/Vis (acetone): *λ*_max_ (*E*_*rel*_) = 477 (1.00), 611 (0.28), 686 (0.11), 761 (0.18) nm; MS (FAB^−^): *m/z* (%) = 606.4 (100) [M^−^ (C_36_H_50_N_2_O_6_^−^)]; HRMS: *m/z* calcd. 606.3674 (C_36_H_50_N_2_O_6_^−^), found 606.3658, Δ = − 1.6 mmu.

### 2,7-Bis(2-hydroxymethyl-2-pentylheptyl)-3,6,8-trioxo-1,2,3,6,7,8-hexahydrobenzo[*lmn*][3,8]phenanthrolin-1-ol radical anion tetrabutylammonium salt (**5d**, C_40_H_58_N_2_O_6_·C_16_H_36_N)

159 mg 2,7-Bis(2-hydroxymethyl-2-pentylheptyl)benzo[*lmn*][3,8]phenanthroline-1,3,6,8-tetraone (**4d**, 0.24 mmol) was allowed to react and purified as was described for 2,7-bis(2-ethyl-2-hydroxymethylbutyl)-3,6,8-trioxo-1,2,3,6,7,8-hexahydrobenzo[*lmn*][3,8]phenanthrolin-1-ol radical anion tetrabutylammonium salt (**5a**). Yield 207 mg (95%) brownish black solid; m.p.: > 300 °C; IR (ATR): $\bar{\nu}$ = 3332 (m, br), 2956 (s), 2930 (m), 2871 (s), 1623 (s), 1586 (s), 1556 (m), 1519 (m), 1487 (w), 1464 (m), 1424 (w), 1400 (w), 1380 (w), 1365 (w), 1298 (m), 1256 (w), 1237 (w), 1189 (w), 1171 (w), 1141 (w), 1089 (w), 1057 (w), 1029 (w), 1012 (w), 979 (w), 958 (w), 932 (w), 879 (w), 833 (w), 812 (w), 759 (w), 739 (w) cm^−1^; ^1^H NMR (400 MHz, acetone-*d*_*6*_): *δ* = 0.98 (t, 12H, ^3^*J* = 7 Hz, R_3_N–(CH_2_)_3_–C*H*_3_), 1.43 (m, 8H, R_3_N–(CH_2_)_2_–C*H*_2_–CH_3_), 1.83 (q, br, 8H, R_3_N–CH_2_–C*H*_2_–CH_2_–CH_3_), 3.45 (t, 8H, ^3^*J* = 8 Hz, R_3_N–C*H*_2_–(CH_2_)_2_–CH_3_) ppm; ESR (acetone): *g *= 2.0040; UV/Vis (acetone): *λ*_max_ (*E*_*rel*_) = 477 (1.00), 611 (0.55), 686 (0.46), 761 (0.51) nm; MS (FAB^−^): *m/z* (%) = 662.4 (100) [M^−^ (C_40_H_58_N_2_O_6_^−^)]; HRMS (C_40_H_58_N_2_O_6_^−^): *m/z* calcd. 662.4300, found 662.4233, Δ = − 6.7 mmu.

### *N*,*N*′-Bis(1-hexylheptyl)-*N*″-(2-hydroxymethyl-2-octyldecyl)benzoperylene-1′,2′,3,4,9,10-hexacarboxylic acid 1′,2′:3,4:9,10-trisimide radical anion tetrabutylammonium salt (**5e**, C_52_H_81_N_2_O_5_·C_16_H_36_N)

47 mg *N*,*N*′′-Bis(1-hexylheptyl)-*N*′-(2-hydroxymethyl-2-octyldecyl)benzoperylene-1′,2′,3,4,9,10-hexacarboxylic acid 1′,2′:3,4:9,10-trisimide (**4e**, 42 μmol) was allowed to react and purified as was described for 2,7-bis(2-ethyl-2-hydroxymethylbutyl)-3,6,8-trioxo-1,2,3,6,7,8-hexahydrobenzo[*lmn*][3,8]phenanthrolin-1-ol radical anion tetrabutylammonium salt (**5a**). Yield 38 mg green solid; UV/Vis (acetone): *λ*_max_ (*E*_*rel*_)= 646 (1.00), 721 (0.59), 863 (0.88) nm.

### 2,9-Bis(2-ethyl-2-hydroxymethylbutyl)anthra[2,1,9-*def*;6,5,10*d′e′f′*]diisoquinoline-1,3,8,10-tetraone radical anion tetrabutylammonium salt (**8a**, C_38_H_38_N_2_O_6_·C_16_H_36_N)

148 mg 2,9-Bis(2-ethyl-2-hydroxymethylbutyl)anthra[2,1,9-*def*;6,5,10-*d*′*e*′*f*′]diisoquinoline-1,3,8,10-tetraone (**7a**, 239 μmol) under argon was dispersed in degassed 0.5 cm^3^ ethanol, treated with 1 cm^3^ degassed distilled water and 1 cm^3^ 30% aqueous NaOH, heated at 50–55 °C, treated with 1 cm^3^ hydroxyacetone (15 mmol), stirred for 10 min, treated with 1.0 g tetrabutylammonium bromide (3.1 mmol) in 1.5 g degassed distilled water, diluted with 10 cm^3^ degassed distilled water, stirred at 0 °C for 10 min, collected by vacuum filtration under argon, washed with degassed distilled water until colorless washings, dried in medium vacuum and then in a counter current of nitrogen over phosphorous pentoxide. Yield 242 mg violet powder; m.p.: > 300 °C; IR (ATR): $\bar{\nu}$ = 3228 (br, m), 1600 (s), 1560 (m), 1541 (s), 1491 (m), 1468 (m), 1437 (m), 1415 (m), 1378 (m), 1358 (m), 1327 (s), 1297 (w), 1228 (m), 1208 (w), 1177 (w), 1145 (m), 1087 (w), 1051 (w), 999 (w), 968 (w), 928 (w), 875 (w), 820 (w), 788 (s), 752 (w), 735 (w), 700 (w), 632 (w), 579 (w) cm^−1^; ^1^H NMR (400 MHz, acetone-*d*_*6*_): *δ *= 0.97 (t, 12H, ^*3*^*J* = 7.3 Hz, N–(CH_2_)_3_–C*H*_3_), 1.37–1.46 (m, 8H, ^*3*^*J* = 7.3 Hz, 7.2 Hz, N–(CH_2_)_2_–C*H*_2_–CH_3_), 1.80 (m, br, 8H, N–CH_2_–C*H*_2_–CH_2_–CH_3_), 3.41–3.45 (t, 8H, ^*3*^*J* = 7.7 Hz N–C*H*_2_–CH_2_–CH_2_–CH_3_) ppm; ESR (acetone): *g* = 2.0038; UV/Vis (acetone): *λ*_max_ (*ε*) = 680 (40,000), 702 (60,000), 711 (60,000), 767 (20,000), 797 (40,000), 957 (20,000) nm (dm^3^ mol^−1^ cm^−1^); MS (FAB^−^): *m/*z (%) = 618.3 (100) [C_38_H_38_N_2_O_6_^−^]; HRMS: *m/z* calcd. 618.2735 (C_38_H_38_N_2_O_6_^−^), found 618.2730, Δ = − 0.5 mmu.

### 2,9-Bis(2-hydroxymethyl-2-propylpentyl)anthra[2,1,9-*def*;6,5,10*-d′e′f′*]diisoquinoline-1,3,8,10-tetraone radical anion tetrabutylammonium salt (**8b**, C_42_H_46_N_2_O_6_·C_16_H_36_N)

164 mg 2,9-Bis(2-hydroxymethyl-2-propylpentyl)anthra[2,1,9-*def*;6,5,10*-d′e′f′*]diisoquinoline-1,3,8,10-tetraone (**7b**, 243 μmol) was allowed to react and purified as was described for 2,9-bis(2-ethyl-2-hydroxymethylbutyl)anthra[2,1,9-*def*;6,5,10*-d′e′f′*]diisoquinoline-1,3,8,10-tetraone radical anion tetrabutylammonium salt (**8a**). Yield 254 mg violet solid; m.p.: > 300 °C; IR (ATR): $\bar{\nu}$ = 3525 (w), 3257 (br, m), 2957 (s), 2932 (s), 2872 (m), 1601 (s), 1542 (s), 1492 (m), 1466 (m), 1436 (w), 1414 (w), 1379 (w), 1359 (m), 1328 (s), 1294 (m), 1226 (m), 1208 (vw), 1177 (vw), 1145 (m), 1103 (w), 1081 (vw), 1057 (m), 1016 (vw), 955 (vw), 928 (w), 879 (w), 850 (vw), 788 (m), 744 (m), 701 (m) cm^−1^; ^1^H NMR (400 MHz, acetone-*d*_*6*_): *δ *= 0.97 (t, 12H, N–(CH_2_)_3_–C*H*_3_), 1.39–1.43 (m, br, 8H, N–(CH_2_)_2_–C*H*_2_–CH_3_), 1.77–1.82 (m, br, 8H, N–CH_2_–C*H*_2_–CH_2_–CH_3_), 3.41–3.45 (m, br, 8H, N–C*H*_2_–CH_2_–CH_2_–CH_3_) ppm; ESR (solid state): *g* = 2.0041; ESR (acetone): *g* = 2.0038; UV/Vis (acetone): *λ*_max_ (*ε*) = 680 (40,000), 702 (60,000), 711 (60,000), 767 (20,000), 797 (40,000), 957 (20,000) nm (dm^3^ mol^−1^ cm^−1^); MS (− p ESI): *m/z* (%) = 674 (100) [M^−^ (C_42_H_46_N_2_O_6_^−^)]; MS (+p ESI): *m/z* (%) = 243 (100) [C_16_H_36_N^+^]; HRMS: *m/z* calcd. 674.3361 (C_42_H_46_N_2_O_6_^−^), found 674.3359, Δ = − 0.2 mmu; calcd. 242.2842 (C_16_H_36_N^+^), found 242.2839, Δ = − 0.3 mmu.

### 2,9-Bis(2-hydroxymethyl-2-propylpentyl)anthra[2,1,9-*def*;6,5,10*-d′e′f′*]diisoquinoline-1,3,8,10-tetraone radical anion tetrabutylammonium salt (**8b**, C_42_H_46_N_2_O_6_·C_16_H_36_N); precipitation of the radical anion with tetrabutylammonium hydrogen sulfate

164 mg 2,9-bis(2-hydroxymethyl-2-propylpentyl)-anthra[2,1,9-*def*;6,5,10-*d′e′f′*]diisoquinoline-1,3,8,10-tetraone (**7b**, 243 μmol) and 1.1 g tetrabutylammonium hydrogensulfate (3.1 mmol) in 1.5 cm^3^ degassed distilled water were allowed to react and purified as was described for 2,9-bis(2-ethyl-2-hydroxymethylbutyl)anthra[2,1,9-*def*;6,5,10*d′e′f′*]diisoquinoline-1,3,8,10-tetraone radical anion tetrabutylammonium salt (**7a**). Yield 254 mg violet solid; IR (ATR): $\bar{\nu}$ = 3525 (w), 3257 (br, m), 2957 (s), 2932 (s), 2872 (m), 1601 (s), 1542 (s), 1492 (m), 1466 (m), 1436 (w), 1414 (w), 1379 (w), 1359 (m), 1328 (s), 1294 (m), 1226 (m), 1208 (vw), 1177 (vw), 1145 (m), 1103 (w), 1081 (vw), 1057 (m), 1016 (vw), 955 (vw), 928 (w), 879 (w), 850 (vw), 788 (m), 744 (m), 701 (m) cm^−1^; UV/Vis (acetone): *λ*_max_ (*ε*) = 680 (40,000), 702 (60,000), 711 (60,000), 767 (20,000), 797 (40,000), 957 (20,000) nm (dm^3^ mol^−1^ cm^−1^).

### 2,9-Bis(2-hydroxymethyl-2-butyloctyl)anthra[2,1,9-*def*;6,5,10-*d′e′f′*]diisoquinoline-1,3,8,10-tetraone radical anion tetrabutylammonium salt (**8c**, C_46_H_54_N_2_O_6_·C_16_H_36_N)

177 mg 2,9-Bis(2-hydroxymethyl-2-butyloctyl)anthra[2,1,9-*def*;6,5,10-*d′e′f′*]diisoquinoline1,3,8,10-tetraone (**7c**, 242 μmol) was allowed to react and purified as was described for 2,9-bis(2-ethyl-2-hydroxymethylbutyl)anthra[2,1,9-*def*;6,5,10*-d′e′f′*]diisoquinoline-1,3,8,10-tetraone radical anion tetrabutylammonium salt (**8a**). Yield 277 mg violet solid; m.p.: > 300 °C; IR (ATR): $\bar{\nu}$ = 3236 (br, m), 2957 (s), 2930 (s), 2871 (m), 1600 (s), 1541 (s), 1492 (m), 1466 (m), 1436 (w), 1413 (w), 1378 (w), 1359 (m), 1328 (s), 1296 (m), 1229 (m), 1208 (w), 1177 (vw), 1145 (m), 1104 (w), 1086 (vw), 1053 (m), 935 (w), 876 (w), 795 (m), 788 (m), 742 (m) 702 (m) 647 (w) cm^−1^; ^1^H NMR (400 MHz, acetone-*d*_*6*_): *δ *= 0.97 (t, 12H, –CH_3_ (C_16_H_36_N^+^)), 1.41–1.43 (m, br, 8H, –C*H*_2_–CH_3_ (C_16_H_36_N^+^)), 1.80–1.82 (m, br, 8H, –C*H*_2_–CH_2_–CH_3_ (C_16_H_36_N^+^)), 3.45–3.46 (t, br, 8H, –CH_2_–N (C_16_H_36_N^+^)) ppm; ESR (acetone): *g* = 2.0037; UV/Vis (acetone): *λ*_max_ (*E*_*rel*_) = 680 (0.62), 701 (0.98), 710 (1.00), 766 (0.26), 796 (0.57), 957 (0.36) nm; MS (FAB^−^): *m/z* (%) = 730.4 (100) [M^−^(C_46_H_54_N_2_O_6_^−^)]; HRMS: *m/z* calcd. 730.3987 (C_46_H_54_N_2_O_6_^−^), found 730.3982, Δ = − 0.5 mmu.

### 2,9-Bis(2-hydroxymethyl-2-pentylheptyl)anthra[2,1,9-*def*;6,5,10*-d′e′f*′]diisoquinoline-1,3,8,10-tetraone radical anion tetrabutylammonium salt (**8d**, C_50_H_62_N_2_O_6_·C_16_H_36_N)

189 mg 2,9-Bis(2-hydroxymethyl-2-pentylheptyl)anthra[2,1,9-*def*;6,5,10-*d′e′f′*]diisoquinoline-1,3,8,10-tetraone (**7d**, 240 mmol) was allowed to react and purified as was described for 2,9-bis(2-ethyl-2-hydroxymethylbutyl)anthra[2,1,9-*def*;6,5,10-*d′e′f′*]diisoquinoline-1,3,8,10-tetraone radical anion tetrabutylammonium salt (**8a**). Yield 346 mg violet solid; m.p.: > 300 °C; IR (ATR): $\bar{\nu}$ = 3250 (br, m), 2955 (s), 2928 (s), 2871 (m), 1600 (s), 1561 (w), 1543 (s), 1488 (m), 1466 (m), 1377 (w), 1360 (m), 1330 (s), 1296 (m), 1229 (m), 1204 (vw), 1145 (m), 1105 (w), 1067 (m), 949 (vw), 878 (w), 788 (m), 741 (m), 702 (m), 646 (w) cm^−1^; ^1^H NMR (400 MHz, acetone-*d*_*6*_): *δ *= 0.98 (t, 12H, –CH_3_ (C_16_H_36_N^+^)), 1.40–1.46 (m, br, 8H, C*H*_2_–CH_3_ (C_16_H_36_N^+^)), 1.82 (m, br, 8H, –C*H*_2_–CH_2_–CH_3_ (C_16_H_36_N^+^)), 3.47–3.48 (t, br, 8H, –CH_2_–N (C_16_H_36_N^+^)) ppm; ESR (acetone): *g *= 2.0037; UV/Vis (acetone): *λ*_max_ (*E*_*rel*_) = 956 (0.36), 796 (0.58), 766 (0.28), 711 (1.00), 701 (0.98), 680 (0.63) nm; MS (− *p* ESI): *m/z* (%) = 786 (50) [M^−^ (C_50_H_62_N_2_O_6_^−^)]; MS (+*p* ESI): *m/z* (%) = 242 (100) [C_16_H_36_N^+^]; HRMS: *m/z* calcd. 786.4631 (C_50_H_62_N_2_O_6_^−^), found 786.4607, Δ = − 2.4 mmu; calcd. 242.2842 (C_16_H_36_N^+^), found 242.2838, Δ = − 0.4 mmu.

### 2,9-Bis(2-hydroxymethyl-2-octyldecyl)anthra[2,1,9-*def*;6,5,10*-d′e′f′*]diisoquinoline-1,3,8,10-tetraone radical anion tetrabutylammonium salt (**8e**, C_62_H_86_N_2_O_6_·C_16_H_36_N)

229 mg 2,9-Bis(2-hydroxymethyl-2-octyldecyl)anthra[2,1,9-*def*;6,5,10*-d′e′f′*]diisoquinoline-1,3,8,10-tetraone (**7e**, 240 µmol) was allowed to react an purified as was described for 2,9-bis(2-ethyl-2-hydroxymethylbutyl)anthra[2,1,9-*def*;6,5,10*d′e′f′*]diisoquinoline-1,3,8,10-tetraone radical anion tetrabutylammonium salt (**8a**). Yield 294 mg violet solid; m.p.: 230 °C; IR (ATR): $\bar{\nu}$ = 3449 (br, w), 3239 (br, w), 2957 (m), 2922 (s), 2852 (m), 1601 (s), 1541 (s), 1490 (m), 1464 (m), 1439 (m), 1361 (w), 1329 (s), 1297 (m), 1228 (m), 1208 (w), 1176 (w), 1147 (m), 1096 (w), 1054 (w), 1024 (w), 940 (w), 912 (w), 876 (w), 788 (s), 740 (s), 724 (w), 703 (m), 648 (w) cm^−1^; ESR (acetone): *g *= 2.0038; UV/Vis (acetone): *λ*_max_ (*E*_*rel*_) = 957 (0.48), 797 (0.63), 767 (0.29), 711 (1.00), 701 (0.97), 680 (0.61) nm; MS (FAB^−^): *m/z* (%) = 954.7 (100) [M^−^ (C_62_H_86_N_2_O_6_^−^)]; HRMS: *m/z* calcd. 954.6491 (C_62_H_86_N_2_O_6_^−^), found 954.6486, Δ = -0.5 mmu.

### 2,9-Bis(1-hexylheptyl)anthra[2,1,9-*def*;6,5,10*-d′e′f′*]diisoquinoline-1,3,8,10-tetraone radical anion tetrabutylammonium salt (**2**, *n *= 2, C_50_H_62_N_2_O_4_·C_16_H_36_N)

180 mg *N*,*N′*-Bis(1-hexylheptyl)anthra[2,1,9-*def*;6,5,10-d′e′f′]diisoquinoline-1,3,8,10-tetraone (**1**, *n *= 2; 0.24 mmol) was allowed to react and purified as was described for 2,9-bis(2-ethyl-2-hydroxymethylbutyl)anthra[2,1,9-*def*;6,5,10*d′e′f′*]diisoquinoline-1,3,8,10-tetraone radical anion tetrabutylammonium salt (**8a**). Yield 197 mg (197 µmol, 82%) violet solid; IR (ATR): $\bar{\nu}$ = 2958 (s), 2924 (vs), 2856 (s), 1582 (vs), 1561 (s), 1524 (s), 1489 (s), 1362 (m), 1318 (s), 1228 (w), 1132 (w), 1100 (w), 782 (w), 742 cm^−1^ (w) cm^−1^; UV/Vis (acetone): *λ*_max_ (*E*_*rel*_) = 680 (0.61), 700 (1.00), 711 (0.92), 766 (0.37), 796 (0.60), 957 (0.43) nm; ESR (acetone): *g *= 2.0037; ESR (solid state): *g *= 2.0041; MS (FAB^−^): *m/z* (%) = 754 (20) [M^−^ (C_50_H_62_N_2_O_4_^−^)]; HRMS: *m/z* calcd. 754.4715 (C_50_H_62_N_2_O_4_^−^), found 754.4756, Δ = 0.5 mmu.

### *N*,*N*′-Bis(1-hexylheptyl)quaterrylene-3,4,13,14-tetracarboxylic acid 3,4:13,14-diimide radical anion tetrabutylammonium salt (**14**, C_70_H_70_N_2_O_4_·C_16_H_36_N)

50 mg *N*,*N*′-Bis(1-hexylheptyl)quaterrylene-3,4,13,14-tetracarboxylic acid 3,4:13,14-diimide (**13**, 50 μmol) was allowed to react and purified as was described for 2,7-bis(2-ethyl-2-hydroxymethylbutyl)-3,6,8-trioxo-1,2,3,6,7,8-hexahydrobenzo[*lmn*][3,8]phenanthrolin-1-ol radical anion tetrabutylammonium salt (**8a**). Yield 63 mg (99%) green solid; IR (ATR): $\bar{\nu}$ = 2957 (m), 2924 (m), 2855 (m), 1643 (m), 1564 (s), 1532 (m), 1504 (m), 1480 (w), 1465 (m), 1376 (m), 1364 (m), 1332 (s), 1292 (w), 1277 (w), 1242 (s), 1203 (w), 1180 (w), 1144 (w), 1104 (w), 1048 (w), 937 (w), 883 (w), 806 (w), 780 (m), 748 (m), 664 (w), 608 (w), 588 (w) cm^−1^; UV/Vis (acetone): *λ*_max_ (*E*_*rel*_) = 1075 (1.00), 1185 (0.16), 1251 (0.30), 1697 (0.20) nm; MS (FAB^−^): *m/z* (%) = 1002.5 (100) [M^−^ (C_70_H_70_N_2_O_4_^−^)]; HRMS: *m/z* calcd. 1002.5341 (C_70_H_70_N_2_O_4_^−^), found 1002.5372, Δ = 3.1 mmu.

### Exemplary procedure for reduction and immediate UV/Vis spectroscopic measurement

2,9-Bis(1-hexylheptyl)anthra[2,1,9-*def*;6,5,10-*d′e′f′*]diisoquinoline-1,3,8,10-tetraone (**1**, *n *= 2; 1.0 mg, 1.3 µmol) was reduced according to **5a** or **8a**, respectively; however, instead of precipitation, a definite small amount of the solution was added under argon to a stock solution consisting of 0.8 cm^3^ DBU (8 mmol), 0.5 cm^3^ hydroxyl acetone (7 mmol), and 1 cm^3^ diacetyl (11 mmol) dissolved in a small amount of spectroscopically pure acetone being quickly completed to 25 cm^3^ by means of acetone and measured against the stock solution (if the time until completing is too long decomposition to brown products will proceed perturbing the measurements).
